# Evaluation of the Tu’Washindi Na PrEP Intervention to Reduce Gender-Based Violence and Increase Preexposure Prophylaxis Uptake and Adherence Among Kenyan Adolescent Girls and Young Women: Protocol for a Cluster Randomized Controlled Trial

**DOI:** 10.2196/55931

**Published:** 2025-04-01

**Authors:** Sarah T Roberts, Alexandra M Minnis, Sue Napierala, Elizabeth T Montgomery, Lina Digolo, Mackenzie L Cottrell, Erica N Browne, Jacqueline Ndirangu, Joyce Boke, Kawango Agot

**Affiliations:** 1 Women's Global Health Imperative RTI International Oakland, CA United States; 2 Department of Epidemiology and Biostatistics School of Medicine University of California, San Francisco San Francisco United States; 3 Evidence and Beyond Nairobi Kenya; 4 UNC Eshelmen School of Pharmacy University of North Carolina at Chapel Hill Chapel Hill, NC United States; 5 Substance Use,Gender, and Applied Research Africa Regional Office RTI International Nairobi Kenya; 6 Impact Research and Development Organization Kisumu Kenya

**Keywords:** adolescent girls and young women, HIV prevention, preexposure prophylaxis, PrEP, adherence, Kenya, intimate partner violence

## Abstract

**Background:**

Adolescent girls and young women constitute a priority population disproportionately affected by HIV, accounting for 25% of annual HIV incidence among people older than 15 years in Kenya. Although oral preexposure prophylaxis (PrEP) is effective in reducing HIV acquisition, its protective benefit has been limited among adolescent girls and young women in sub-Saharan Africa because of low uptake, adherence, and persistence. Intimate partner violence (IPV) and relationship power inequities are widespread among adolescent girls and young women and contribute to higher HIV incidence and lower PrEP use. Interventions are needed to support sustained PrEP use among adolescent girls and young women by addressing IPV and relationship dynamics.

**Objective:**

This study aims to test the effectiveness of Tu’Washindi na PrEP (“We are Winners with PrEP”), a multilevel community-based intervention, to increase uptake and adherence to PrEP and reduce IPV among adolescent girls and young women in Siaya County, Kenya.

**Methods:**

The Tu’Washindi na PrEP intervention was co-designed by our team and adolescent girls and young women using participatory methods and includes 3 components delivered over 6 months: an 8-session, empowerment-based support club for adolescent girls and young women, community sensitization targeted toward male partners, and PrEP education events for couples. The intervention will be evaluated using a cluster randomized controlled trial across 22 administrative wards in Siaya County, Kenya, enrolling 72 adolescent girls and young women per ward (total N=1584). The primary objectives are to test the effectiveness of the intervention on PrEP uptake and adherence immediately after delivery (month 6 after enrollment) and 6 months later (month 12). As secondary objectives, we will test the intervention effect on IPV. A rigorous process evaluation will explore mechanisms of change, contextual factors, and implementation considerations to inform future refinement and scale-up, using programmatic data, participant questionnaires, and qualitative interviews with participants and intervention providers.

**Results:**

Data collection started in September 2022. As of December 2024, enrollment has been completed in 16 of the 22 study wards, with 72.6% (1150/1584) of participants enrolled. We anticipate that data collection will be completed in May 2026 and results will be available by mid-2027.

**Conclusions:**

The study builds directly on our promising formative and pilot research to develop the evidence base for this youth-designed, multilevel HIV prevention intervention. If effective, Tu’Washindi will be ideally positioned for sustainable integration into existing youth-focused programming to expand and support PrEP use in this priority population.

**Trial Registration:**

ClinicalTrials.gov NCT05599581; https://www.clinicaltrials.gov/study/NCT05599581

**International Registered Report Identifier (IRRID):**

DERR1-10.2196/55931

## Introduction

### Background

Optimizing the effectiveness of proven biomedical prevention interventions is critical to decreasing the high incidence of HIV among adolescent girls and young women aged 15 to 24 in sub-Saharan Africa, a priority population for HIV prevention. More than 1 in 4 new HIV infections in this region are among adolescent girls and young women [1], who are over twice as likely to acquire HIV as their male counterparts [2]. Oral preexposure prophylaxis (PrEP) is an effective, female-initiated biomedical HIV prevention intervention, but its protective benefit has been limited among adolescent girls and young women across sub-Saharan Africa by challenges to uptake, adherence, and persistence [3-17]. In western Kenya, a study of adolescent girls and young women attending family planning clinics found that 76% met PrEP eligibility criteria, but only 4% initiated PrEP [16], and the proportion of adolescent girls and young women persisting with PrEP for 3 months postinitiation ranged from 5% to 37% across studies [7,10,11,15,17]. In samples of PrEP users at an average of 6 months postinitiation, only 4% to 8% had tenofovir diphosphate (TFVdp) levels suggesting high adherence [4-6]. The public health impact of PrEP will be determined by concurrent interventions that address critical barriers to uptake and adherence.

Adolescent girls and young women who live in a context of heightened gender inequality and risk of intimate partner violence (IPV) represent a large subpopulation who are uniquely vulnerable to HIV infection. IPV is widespread among adolescent girls and young women in western Kenya: 19% reported experience of sexual IPV in the past year, 25% reported physical IPV, and 34% reported emotional IPV [18-20]. Experience or fear of IPV in sexual relationships is associated with having limited relationship power [20,21] and with 28% to 55% higher HIV incidence in adolescent girls, young women, and adult women [21-23]. Studies have found that IPV and other partner-related social harms are associated with 1.5- to 2.5-fold higher risk of poor adherence to oral PrEP and the dapivirine ring for HIV prevention. Similar to HIV treatment and contraception [19,20,24-29], IPV and relationship inequality introduce barriers to uptake of and adherence to PrEP at multiple levels. At the individual-level, restricted access to information and health services, poor negotiation skills, low self-efficacy, and fear of violence or relationship dissolution hinder adoption of health-promoting behaviors [30-32]. Dynamics at the partner level, such as poor communication and low decision-making power, limit disclosure about PrEP use and reduce partner support, which can impact adherence [33-35]. These challenges are further compounded at the community level by a lack of PrEP awareness, inequitable gender norms, and stigma associated with taking PrEP, which contribute to partner and community opposition to female PrEP use [36-39]. A multilevel context-specific intervention to address barriers to PrEP use is needed to ensure that adolescent girls and young women experiencing relationship inequality and IPV benefit from this effective biomedical prevention strategy.

In response, our intervention, Tu’Washindi na PrEP (We are winners with PrEP), was designed in close partnership with local adolescent girls and young women and incorporates strategies targeted to address relationship dynamics and IPV at multiple levels [40,41]. This intervention has 3 components: an empowerment-based support club for adolescent girls and young women, PrEP education events for couples offered in the context of a health fair (Buddy Days), and community sensitization about PrEP targeted toward adolescent girls and young women’s partners [40]. The pilot cluster randomized controlled trial (cRCT) of Tu’Washindi at 6 Determined, Resilient, Empowered, AIDS-free, Mentored and Safe (DREAMS) spaces demonstrated feasibility, high acceptability, implementation with fidelity, and promising effects on PrEP and IPV outcomes [42]. PrEP uptake and adherence were both approximately twice as high in the intervention arm as in the control arm (*P*<.05) [43]. Although the adherence was still lower than desired in the intervention arm, with Wisepill openings on 25% of days on PrEP, they still represented substantial improvement over programmatic outcomes [6,17]. We also observed less frequent or severe IPV among intervention arm participants [43].

Because our pilot findings suggested that Tu’Washindi shows promise as an acceptable intervention that can be implemented with fidelity to promote PrEP uptake and adherence among adolescent girls and young women without concomitant increases in IPV, we designed a fully powered effectiveness trial to determine whether these gains translate to increases in biomarker measures of PrEP adherence and whether the intervention can reduce IPV risk.

### Study Objectives

The objective of this study is to test the effectiveness of the multilevel Tu’Washindi intervention to increase effective PrEP use and reduce IPV among Kenyan adolescent girls and young women by addressing relationship dynamics and partner opposition to PrEP. A process evaluation will assess the implementation processes and theorized mechanisms of change influencing intervention effectiveness and identify implementation challenges and strategies to facilitate future scale-up in programmatic settings to maximize public health impact. Study findings will contribute to the limited evidence base for effective PrEP adherence interventions to reduce HIV acquisition in this priority population.

## Methods

### Design

**Figure 1:**
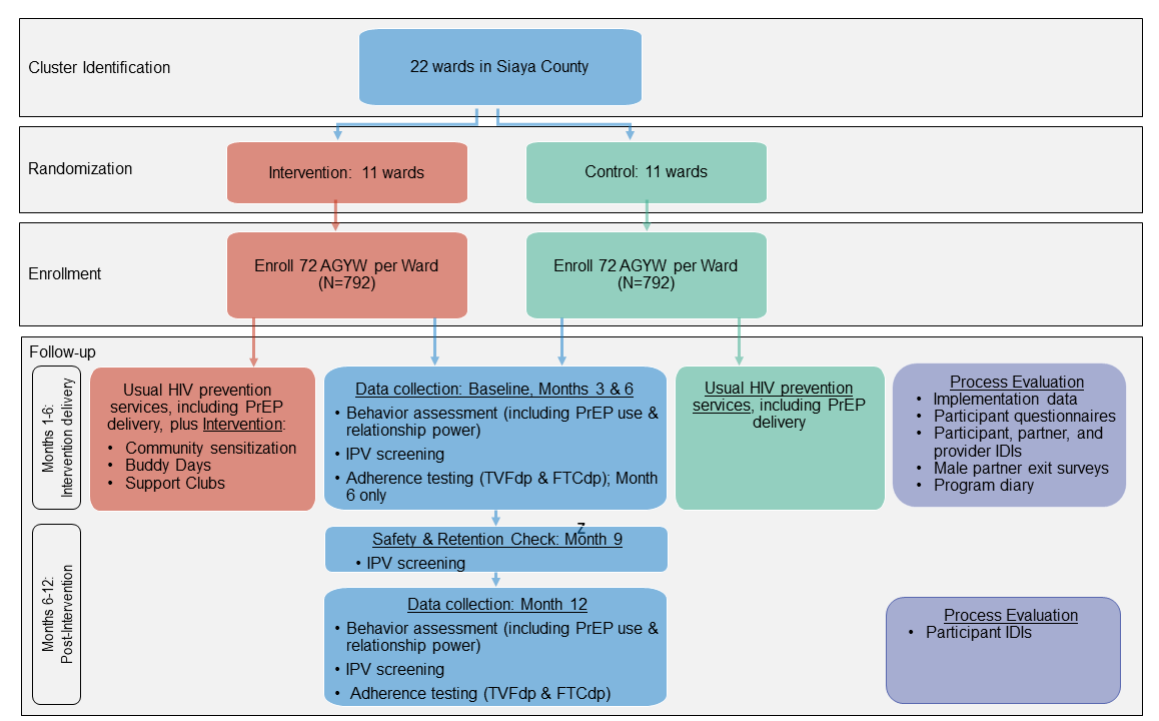
An overview of the study design. AGYW: adolescent girls and young women; FTCtp: emtricitabine triphosphate; IDI: in-depth interview; IPV: intimate partner violence; PrEP: preexposure prophylaxis; TFVdp: tenofovir diphosphate.

This study has a 2-arm parallel cRCT design (Figure 1). We have randomized 22 administrative wards in a 1:1 ratio and aim to enroll about 72 adolescent girls and young women from each (total N=about 1584) to receive either the Tu’Washindi intervention plus usual HIV prevention services, or usual HIV prevention services alone. Because of the urgent need for interventions to support PrEP use among adolescent girls and young women in a real-world context, the intervention is delivered outside of DREAMS to evaluate its impact on adolescent girls and young women accessing PrEP from Ministry of Health (MoH) facilities. This pragmatic study design enables us to determine effectiveness of the intervention when layered onto ongoing county-wide HIV prevention programming [44]. After informed consent and baseline data collection, the Tu’Washindi intervention is implemented in each intervention cluster while the control cluster continues with usual HIV prevention services. The duration of study participation is 12 months, with data collection visits at intervention midline (study month 3), intervention endline (study month 6), and at 6 months postintervention (study month 12). A prospective process evaluation is being conducted to characterize intervention implementation, explore theorized mechanisms of change, and capture contextual factors influencing study outcomes. The protocol was written following the 2013 SPIRIT (Standard Protocol Items: Recommendations for Interventional Trials) guidelines (Multimedia Appendix 1).

### Study Setting

Siaya County is located in the former Nyanza Province in western Kenya, along the shores of Lake Victoria. The county is primarily periurban and rural, and agriculture and fishing are the main economic activities [45]. Siaya County has the second highest HIV incidence in Kenya (2.5% per year); it is the site of 12% of new HIV infections but comprises just 2% of the national population [46]. In addition, the former Nyanza Province has the highest prevalence of gender-based violence in Kenya: 22% of women aged 15 to 49 have reported sexual violence and 56% have reported physical violence at least once since the age of 15 [18].

### Study Organization

The Tu’Washindi study leadership is comprised of investigators from RTI International and Impact Research and Development Organization (IRDO) and meets monthly. This group is ultimately responsible for the design and conduct of the trial, including intervention updates, protocol preparations and revisions, and protocol and safety monitoring. The trial management committee meets weekly, and it is responsible for the day-to-day conduct of the trial. It includes the principal investigator (PI) and site PI, project director and project coordinator, intervention leads, and quantitative and qualitative data management teams. The University of North Carolina Center for AIDS Research Clinical Pharmacology and Analytical Chemistry Core is responsible for analysis of the dried blood spot (DBS) samples. The study is further supported by a 5-member national-level technical advisory committee who meet annually and advise on design considerations to ensure a smooth transition from research to implementation if the intervention is effective; a 15-member youth advisory board that meets quarterly and provides feedback on study design and methods, data collection tools, and recruitment and retention of study participants; and a 12-member stakeholder advisory board made up of PrEP technical staff from MoH and other delivery partners, IPV referral agency representatives, and other community leaders that meets twice yearly to advise on effective integration of the study into existing structures and on linkage to PrEP and other referral services.

### cRCT Component

#### Site Selection and Randomization

The study clusters comprise 22 of the 30 administrative wards in the county. The 30 wards were categorized into 5 strata based on community type (ie, periurban, rural fishing, or rural nonfishing) and whether the proportion of girls on PrEP is above or below the median, using MoH programmatic data. (Although the geography and PrEP use criteria define 6 strata, only 1 ward in the county fell within the urban, low PrEP use stratum. Because it was not possible to create a pair of intervention and control wards in this stratum, it was excluded.) The 22 study wards were purposively selected to ensure an even number of wards within each stratum and a balanced distribution of wards across strata. Randomization was completed by RTI statisticians once the 22 study wards are selected and placed into strata. Within each stratum, wards were randomly allocated to the intervention or control arms in a 1:1 ratio. After randomization, we created pairs of intervention and control wards within each stratum and determined the order in which they would be enrolled in the study based on the location of each ward, to minimize the risk of contamination while ensuring the feasibility of implementation. Subsequently, one intervention and control site pair were randomly selected from each stratum for inclusion in the qualitative component. Allocation was revealed to the investigators before community mapping (see the Recruitment and Screening section) and to the participants after their enrollment into the study.

#### Eligibility Criteria

To be eligible, potential participants must be females aged 15 to 24, currently in a sexual relationship with a male partner for at least 1 month, and vulnerable to HIV per a modified version of the DREAMS eligibility screening tool. To reflect the population most likely to participate in this intervention in a programmatic setting, they must be either taking PrEP or interested in taking PrEP (ie, she thinks that she would benefit from PrEP but is not currently taking it). In addition, they must be residents of the applicable study ward; willing and able to attend support clubs; willing and able to provide adequate contact information; fluent in English, Dholuo, or Kiswahili; and able and willing to provide informed consent (or assent and parental consent for nonmature minor participants aged 15 to 17). Potential participants are excluded if they are living with HIV (by self-report); planning any long-term travel or relocation in the next 12 months; or have any condition that the site PI or designee determines would preclude participation.

Individuals who do not meet the criteria for participation in this trial (ie, screen failure) because of criteria that are likely to change over time may be rescreened. Examples include not being resident of the ward or not being interested in taking PrEP.

#### Recruitment and Screening

Recruitment occurs simultaneously in each pair of wards before moving on to the next pair and focuses on the catchment areas of 2 to 4 health facilities in each ward. Before recruitment, community mapping is conducted to identify key stakeholders, referral resources, locations where adolescent girls and young women gather, and locations where men gather. Recruitment takes place at venues identified through community mapping, including health facilities, schools, churches, DREAMS Safe Spaces, youth groups, and community markets. To ensure that the study findings are generalizable to adolescent girls and young women across the 15- to 24-year age range, we aim to enroll at least 25% of the study participants from the 15- to 19-year age group who have lower rates of PrEP use but have similar HIV incidence to the 20- to 24-year age group [47,48]. Attendees interested in the study are invited to meet with the research assistant after the recruitment meeting to discuss questions, obtain additional information, and schedule an appointment for informed consent, eligibility screening, and, if eligible, for enrollment.

#### Retention

Once a participant is enrolled in the study, the study team makes every reasonable effort to retain her to minimize bias associated with loss to follow-up. To ensure high retention, IRDO tracks retention rates and follows standard study operating procedures detailing strategies, such as clear explanation of the visit schedule at all visits, collection of detailed locator information with multiple means to contact participants, appropriate and timely visit reminders, offering weekend hours for study visits, and conducting immediate follow-up on missed visits through phone, home or other off-site visits.

#### Intervention and Control Conditions

##### Intervention Design

**Figure 2:**
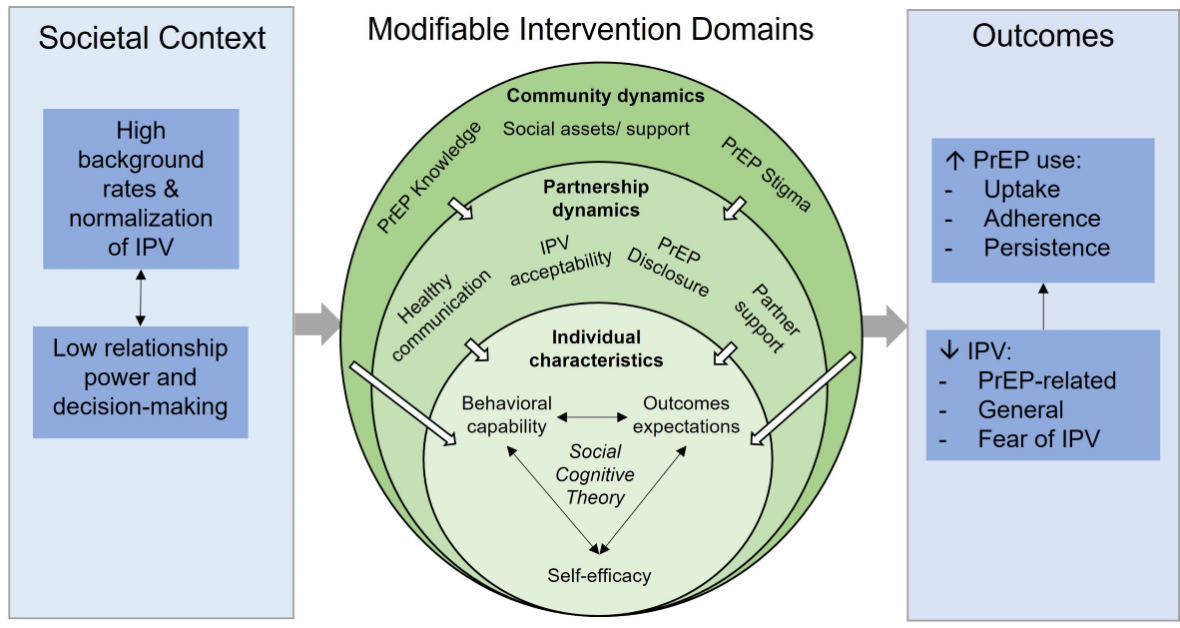
The conceptual framework; adapted from Pettifor et al [51] and Mathur et al [50]. IPV: intimate partner violence; PrEP: preexposure prophylaxis.

Tu’Washindi was developed by our team using a participatory process with adolescent girls and young women and service providers in Siaya County to ensure that it was responsive to adolescent girls and young women’s stated needs for PrEP support in the context of their relationships [40]. The intervention components are informed by social cognitive theory [49] in combination with a socioecologic framework for PrEP introduction [50] to address factors at the individual, partnership, and community levels that influence adolescent girls and young women’s response to IPV and their PrEP use (Figure 2 [50,51]).

Social cognitive theory informs support club activities to enhance individual-level behavioral capability (eg, knowledge and skills to use PrEP and IPV safety planning), improve outcomes expectations (eg, that PrEP use will prevent HIV without threats to safety or relationship security), and increase self-efficacy to use PrEP safely and consistently. At the partnership level, support clubs also aim to develop skills for healthy communication and PrEP disclosure. Drama activities are integrated into each session to allow participants to practice these skills and build self-efficacy. Adolescent girls and young women follow suggested storylines from the intervention manual, developed by youth in the formative phase [40], to create and enact plays about overcoming partner-related barriers to PrEP use, including one to be performed at the Buddy Days. Buddy Days also work at this level to facilitate disclosure and build male partner support for PrEP use by engaging couples in discussions about PrEP, healthy relationships, and promoting HIV prevention as part of family well-being. To increase men’s receptiveness to information provided at the Buddy Days or from their female partners, community sensitization events deliver accurate information about PrEP (eg, safety, effectiveness, and eligibility), discuss common myths and misperceptions, and encourage men to support their partners’ PrEP use [53]. At the community level, the community sensitization activities aim to increase PrEP knowledge, reduce PrEP stigma, and reduce the acceptability or normalization of IPV as a response to adolescent girls and young women’s PrEP use. Support clubs aim to increase social assets, including peer support for PrEP use and access to IPV resources in the community. Table 1 illustrates the intervention’s alignment with the conceptual framework.

**Table 1:** Mapping of intervention components to conceptual framework.

Level and domain			Community sensitization		Buddy Days		Support clubs
**Individual**							
	Behavioral capability					✓	
	Outcomes expectations			✓		✓	
	Self-efficacy					✓	
**Partnership**							
	Healthy communication			✓		✓	
	Reduce intimate partner violence acceptability	✓		✓		✓	
	Disclosure of preexposure prophylaxis use			✓		✓	
	Partner support or acceptance	✓		✓			
**Community**							
	Preexposure prophylaxis knowledge	✓		✓			
	Stigma	✓					
	Social assets					✓	

##### Intervention Delivery

Before delivery, the intervention manual and training tools were updated to reflect lessons learned during the pilot and the change to implementation outside of DREAMS. Because age restrictions on PrEP in DREAMS did not permit us to pilot the intervention with adolescent girls aged 15 to 17 years, we pretested the activities with 12 to 15 adolescent girls in this age range over a 1-month period to obtain feedback on comprehension and relevance of the content and how best to support participation.

Delivery takes place in 4 phases, with activities occurring concurrently in 2 wards during phase 1 and in 3 wards during phases 2 to 4. In each ward, the intervention is conducted over a 6-month period with intervention implementation beginning about 1 week after enrollment concludes. Each new phase of the intervention commences immediately after the activities in the previous phase are concluded. This phased approach minimizes staff burden and allows for close monitoring of implementation to identify and correct problems early on.

The intervention activities include sensitization of MoH clinics, intervention staffing and training, community sensitization sessions, Buddy Days, support clubs, and monitoring and quality assurance.

For sensitization of MoH clinics, before study initiation in each intervention ward, we collaborate with the facility in-charges from each of the 2 to 4 health facilities selected for study recruitment to identify PrEP delivery staff and schedule a half to one day training to introduce the study and discuss relationship-related challenges to PrEP use, with the goal of ensuring that the counseling support offered at the clinics complements the messages provided through the intervention. We also review PrEP adherence and couples’ counseling and disclosure approaches to more comprehensively address adherence challenges commonly experienced by adolescent girls and young women.

Intervention staffing and training within each ward is delivered by a set of 2 to 4 mentors (ie, women slightly older than the participants who have completed at least some secondary education and are rooted in their communities), an experienced male community organizer, and clinicians and counselors with experience in PrEP provision to adolescent girls and young women (hereafter “intervention providers”). Staff supervisors train each cadre over 1 to 3 days to review the intervention manual and to practice the required activities.

Community sensitization sessions are conducted by the community organizer approximately weekly over the first 3 months of the intervention period, lasting about 60 minutes to 90 minutes each. Sessions occur in places where adolescent girls and young women’s male partners are known to gather [54]. Content focuses on PrEP information and dispelling myths and rumors. When time allows, additional content on healthy relationship communication is also included. Clinicians may attend larger sessions to support the organizer.

Buddy Days take place around month 3 of the intervention period, with about 2 to 3 events per ward to maximize participation. The sessions are cofacilitated by the community organizer and clinician and include provision of information about PrEP; facilitated discussion of reasons to support adolescent girls and young women’s PrEP use, healthy relationship communication, and acceptability of IPV; and an interactive drama, designed and performed by volunteer Tu’Washindi participants based on story guides in the intervention manual, to illustrate real-world partner-related challenges to PrEP use. The cofacilitators foster audience engagement both during and after the performance by soliciting reflections on the drama, discussions of alternate storylines, and experiences from audience members [55]. To encourage and destigmatize participation, Buddy Days are open to all community members and offer health services, such as HIV testing services and screening for hypertension and diabetes. In addition, all adolescent girls and young women and their partners who attend the Buddy Days together as a couple receive a small basket of foodstuffs worth about US $5 as an incentive.

Support clubs meet for approximately eight 2-hour sessions over the 6-month intervention period, facilitated by the mentor or by 1 or more participants with mentor support. About 3 to 4 support clubs are offered at varying times and locations to maximize convenience and maintain a small enough number of attendees (18-24) to build community and trust. The support clubs aim to be youth-friendly and nonjudgmental, with an emphasis on ground rules and confidentiality, and support for PrEP uptake and adherence are integrated throughout. Each interactive, participatory session follows the same basic format: a check-in and activity to build community; recap of the previous session; a new topic related to PrEP and relationships, led by the mentor, clinician, or counselor (Table 2); drama activities; and a closing activity to reflect on lessons learned and build confidence and support.

**Table 2:** Sample support club topics and coleaders.

Session and topic	Leaders
Session 1: PrEP^a^ information	Mentor and clinician
Session 2: disclosure	Mentor and counselor
Session 3: undisclosed PrEP use	Mentor and clinician
Session 4: healthy relationships	Mentor and counselor
Session 5: IPV^b^ and PrEP use	Mentor and counselor
Session 6: open session^c^	Mentor
Session 7: open session^c^	Mentor
Session 8: future goals and closing ceremony	Mentor

^a^PrEP: preexposure prophylaxis.

^b^IPV: intimate partner violence.

^c^Participants’ choice of topics.

Monitoring and quality assurance involves closely monitoring intervention delivery for quality and fidelity. Staff supervisors will observe at least 15% of community sensitization activities, 100% of Buddy Days, and at least 20% of support clubs using activity-specific observation forms to document assessments of fidelity (ie, adherence to the intervention manual), quality of facilitation (eg, facilitator maintains focus and nonjudgmental delivery), participant engagement, and any factors that may have affected implementation. Intervention staff receive feedback and coaching after each observed session. Findings will inform staff retraining and implementation refinement for subsequent phases. For example, training of intervention providers may be bolstered if gaps are identified.

##### Usual Care Services

Participants in the intervention and control clusters will have access to a range of evidence-based HIV prevention services offered in the county, including Global Fund–supported services, such as HIV testing services, syndromic management of sexually transmitted infections, family planning and postviolence care services; ongoing DREAMS programming; and PrEP [56]. Participants in both arms receive information about available services and are referred to these services upon request. PrEP is available to adolescent girls and young women aged ≥15 at >100 MoH facilities throughout the county [57], and all the participants who choose to use PrEP in the study can access it through these MoH facilities. In keeping with our study design, this choice of usual services as the comparator condition is realistic, ethical, and relevant [58] because it represents a wide range of evidence-based services to reduce HIV risk. However, PrEP uptake and persistence have been suboptimal in this context, indicating that the intervention is warranted, and that the comparator condition is not sufficiently robust to preclude our ability to detect an effect [59].

#### Data Collection

Data collection visits occur simultaneously in each pair of wards at enrollment, and months 3, 6, and 12 (Table 3). At enrollment, interviewer-administered questionnaires are used to assess sociodemographics, relationship characteristics, IPV, behavioral HIV risk factors, and history of PrEP use. Measures are repeated in follow-up questionnaires to document changes in relevant characteristics, attitudes, and behaviors.

DBS samples are collected via fingerprick at months 6 and 12 from all participants who report initiation or continuation of PrEP since the last visit. For participants who report discontinuation of PrEP, reasons for discontinuation are recorded, but DBSs are not collected. Due to storage and transport challenges, DBS samples are also not collected for participants who have relocated outside the county and have follow-up visits conducted by phone or at a remote location. DBS samples are stored in freezers at the IRDO laboratory and then shipped to the laboratory at the University of North Carolina Center for AIDS Research Clinical Pharmacology and Analytical Chemistry Core for quantification of drug concentrations by liquid chromatography-tandem mass spectrometry technology [60].

**Table 3:** Participant timeline.

Procedures			Month 0 (BL^a^)		Month 1		Month 2		Month 3 (3 MFU^b^)		Month 4		Month 5		Month 6 (6 MFU)		Month 7		Month 8		Month 9		Month 10		Month 11		Month 12 (12 MFU)
Assess and confirm eligibility			✓																								
Informed consent			✓																								
Enrollment			✓																								
Locator information and update			✓						✓						✓						✓						✓
**Quantitative data collection**																											
	Sociodemographic and behavioral characteristics	✓						✓						✓												✓	
	PrEP^c^ use history	✓						✓						✓												✓	
	IPV^d^ history	✓						✓						✓												✓	
	Hypothesized mechanisms of action^e^	✓						✓						✓												✓	
	Laboratory assessment (dried blood spots)													✓												✓	
**Tu’Washindi intervention**																											
	Community sensitization session (weekly)			✓		✓		✓																			
	Buddy Days							✓																			
	Support clubs (once or twice per month)			✓✓^f^		✓✓^f^		✓		✓		✓		✓													
**Qualitative data collection**																											
	In-depth interviews (subset of participants)													✓												✓	
**Safety monitoring**																											
	Standardized assessments							✓						✓												✓	
	Short in-person or telephone contact visit																			✓							
	Reporting of IPV, injuries, social harms, or other relevant concerns			✓		✓		✓		✓		✓		✓		✓		✓		✓		✓		✓		✓	

^a^BL: baseline appointment.

^b^MFU: month follow-up appointment.

^c^PrEP: preexposure prophylaxis.

^d^IPV: intimate partner violence.

^e^Also part of process evaluation.

^f^Two support clubs per month.

### Process Evaluation Component

#### Overview

**Figure 3:**
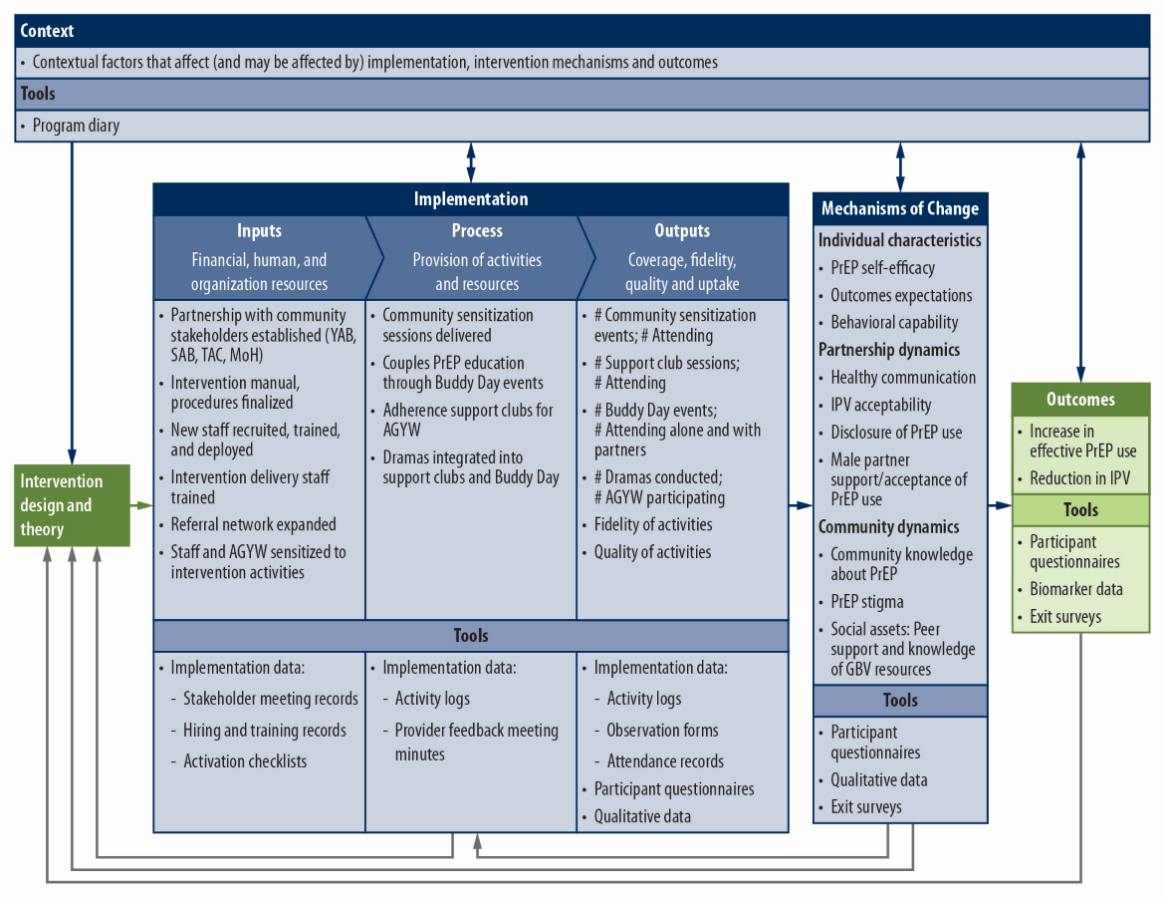
Process evaluation framework and key measures. Adapted from the Medical Research Council guidelines for process evaluation of complex interventions [60]. IPV: intimate partner violence; MoH: Ministry of Health; PrEP: preexposure prophylaxis; SAB: Stakeholder Advisory Board; TAC: Technical Advisory Committee; YAB: Youth Advisory Board.

The mixed methods process evaluation uses implementation data; quantitative adolescent girls and young women participant questionnaires; exit surveys with men who attend intervention activities; qualitative in-depth interviews (IDIs) with adolescent girls and young women participants, their male partners, and intervention providers; and a project diary. Our process evaluation framework (Figure 3 [61]) highlights the key evaluation components, relationships between them, and key measures and data sources [62-65].

#### Participants and Eligibility

Eligibility criteria for adolescent girls and young women participants are described in the Eligibility Criteria section. Participants in exit surveys or male partner IDIs must be males aged ≥15 years. In addition, the exit survey participants must have attended a study Buddy Days or community sensitization event and have been present for at least half of the event, while IDI participants must be a sexual partner of the adolescent girls and young women clinical trial participant and that adolescent girls and young women participant must have provided permission for the study staff to contact the male partner. Intervention providers must be aged ≥18 years and have been involved in delivering Tu’Washindi intervention activities. In addition, all participants must be fluent in English, Dholuo, or Kiswahili; willing and able to provide informed consent; and not have any condition that the site PI or designee determines would preclude participation.

#### Participant Selection and Recruitment

##### Exit Surveys

At all intervention sites, all men who attend a Buddy Days event as a couple are approached while waiting for incentive distribution and asked to complete a survey. A research assistant approaches about 20% of men who attend community sensitization events and asks them to complete survey after the event.

##### In-Depth Interviews

For each of the 5 intervention and control ward pairs randomly selected for qualitative research, we use data captured at month 3 to purposively select a subset of adolescent girls and young women participants (n=30; 4 per intervention ward and 2 per control ward) who are behaviorally eligible for PrEP, per self-report, and have attended at least one support club meeting (intervention wards only). We aim to ensure representation of PrEP users and nonusers and those with and without a history of IPV. Participants who are selected for IDIs are contacted by a research assistant before their month 6 visit, informed that they have been selected, and invited to participate in the IDI.

On the basis of the information provided by the adolescent girls and young women at the month 6 visit, we purposively select male partners (n=20; 4 per intervention ward) for IDIs to represent a range of exposure to the Buddy Days and community sensitization activities.

Male partners are only contacted with the permission of the adolescent girls and young women participants, who provide contact information. A research assistant then contacts the male partner and invites him to participate in the IDI. Study staff do not disclose the PrEP use status of adolescent girls and young women participants to any partner whom they recruit for the IDIs.

Intervention providers are purposively selected for IDIs by the study coordinator to represent a variety of job functions, including mentors, community organizers, counselors, and clinicians (n=20, 4 per intervention ward).

#### Data Collection

Implementation data includes staff hiring and training records; minutes from youth advisory board, stakeholder advisory board, and technical advisory committee meetings; and study activation checklists to track activities, materials, and systems required for successful implementation, such as community introductions, final intervention manuals, and vetted lists of referral agencies. Providers maintain activity logs and attendance records for each meeting or event, and supervisors record minutes from feedback and coaching meetings. Quality and fidelity of intervention activities is documented on observation forms as described above.

Participant questionnaires are administered to adolescent girls and young women participants at each visit to assess mechanisms of change (for all participants) and perceived intervention quality (intervention arm) or potential contamination (control arm). The hypothesized mechanisms of change include PrEP self-efficacy and outcome expectations, PrEP knowledge, healthy communication, disclosure of PrEP use to male partner and partner reaction, community knowledge of PrEP, PrEP stigma, and social assets.

Exit surveys are brief, anonymous, quantitative, interviewer-administered questionnaires that gather information on men’s views of PrEP, their willingness to support their partner’s PrEP use, and feedback on intervention activities.

IDIs are conducted in a private location and facilitated by trained female interviewers following a semistructured guide as follows:

- Serial IDIs with adolescent girls and young women participants are conducted at 2 time points (months 6 and 12). Key topics include relationship quality and experiences of IPV, patterns of PrEP use and barriers and facilitators to PrEP access and use, experiences with the intervention, and perceptions of how the intervention has affected the hypothesized mechanisms of change and study outcomes.
- Male partner IDIs take place at the end of the intervention period and explore men’s experiences with the intervention. Interviews also aim to identify mechanisms through which their attitudes toward PrEP were shifted, or reasons why they were not.
- Intervention provider IDIs are conducted at the end of intervention delivery to elicit their perceptions of the adequacy of inputs (eg, training and resources), fidelity and quality of delivery, and challenges and perceived benefits to delivering each intervention component.

A program diary is maintained by the study coordinator to record external events (eg, elections, police activity, festivities, and health campaigns) and internal events (eg, increased funding or supply shortages) that may affect intervention delivery or study outcomes. Before intervention implementation in each ward, the study coordinator identifies a set of sources, including study staff, health facility personnel, community members or others who are well-positioned to provide this contextual information, and contacts them at regular intervals to record any updates.

### Data Management

Most questionnaire data are collected on tablets using REDCap (Research Electronic Data Capture; Vanderbilt University) or collected on paper forms and entered into REDCap at the study offices, with hard copies securely stored at the field site [66,67]. RTI implements a quality assurance plan, including routine generation of quality control reports by the data manager and communication of findings for resolution. IDIs are audio-recorded, with debriefing reports completed immediately after each interview and quickly shared with the study team to highlight preliminary findings. Interviews are translated and transcribed at the study site. Electronic copies of forms are securely transmitted to RTI and reviewed for quality control, with weekly query resolutions.

### Sample Size Considerations

The study is powered to identify an absolute difference of 15% between intervention and control arms for the primary outcomes: the proportion with effective PrEP use at month 6 and month 12. Power calculations are conducted using simulations based on the logistic model with normally distributed ward (cluster) effects and assuming 11 wards per treatment arm with type 1 error of 0.025 to account for separate analyses at each of 2 time points. With 60 participants per ward, we will have ≥80% power to detect a 15% difference in the outcome as long as the SD for ward variation is ≤10% and the level of effective PrEP use in control wards remains <50% (far higher than observed in recent trials and demonstration projects). To allow for up to 20% loss to follow-up, our total sample size will be 1584 participants: 72 adolescent girls and young women in each of the 22 wards (11 wards per arm).

### Data Analysis: Clinical Trial

#### Outcomes and Hypotheses

The primary outcomes of the study will be the proportion of study participants with effective PrEP use, defined in Table 4, evaluated immediately postintervention, at month 6, and at month 12 to gauge the persistence of intervention effect. TFVdp levels will be dichotomized by Clinical Pharmacology and Analytical Chemistry Core laboratory specialists to delineate between consistent (≥4 doses per week) versus inconsistent (<4 doses per week) adherence [68]. The aim 1 hypothesis is that the proportion of participants with effective PrEP use at (a) month 6 and (b) month 12 will be *higher* in the intervention versus control arm.

**Table 4:** An overview of key measures for the cluster randomized controlled trial, collected at enrollment and months 3, 6, and 12, unless otherwise noted.

Measure		Method
**Primary outcomes**		
	Effective PrEP^a^ use (biomarker assessment)	Percentage of all study participants with biomarker TFVdp^b^ levels indicating consistent PrEP use (≥4 doses per week) for the past 2 months. [68,69]. Represents a composite outcome of PrEP initiation and high execution to capture these key steps in the HIV prevention continuum. Collected at months 6 and 12 only.
**Secondary outcomes**		
	IPV^c^ prevalence	Percentage of participants reporting IPV since the last study visit, measured with the WHO VAWI^d^ [70] and classified by the STRIVE consortium definition [71]: any act of severe physical or sexual violence or ≥2 acts of moderate physical violence.
	IPV severity	Percentage of participants reporting any sexual or severe physical IPV since the last study visit, measured with WHO VAWI [70].
	IPV intensity	Continuous score calculated from the number of specific violent acts reported and the reported frequency of each act (0=never; 1=once; 2=a few times; and 3=often) since the last study visit, measured with WHO VAWI [70].
**Exploratory outcomes (subcomponents or alternate measures of primary and secondary outcomes)**		
	PrEP uptake	Percentage of participants initiating PrEP *among those not on PrEP at baseline*, self-reported and validated by Ministry of Health records.
	PrEP execution	Percentage of participants with TFVdp levels indicating consistent use *among those reporting current PrEP use.*
	PrEP persistence	Percentage of participants with any detectable TFVdp *among those who ever initiated PrEP.* Participants who self-report discontinuation will be categorized as having undetectable TFVdp levels.
	Recent PrEP use (biomarker)	Percentage of all study participants with biomarker FTCtp^e^ levels indicating consistent PrEP use (≥4 doses per week) for the past 2 wk. Collected at months 6 and 12 only.
	IPV type	Percentage of participants reporting each IPV type (physical, sexual, emotional, and economic) since the last visit, per WHO VAWI [70].

^a^PrEP; preexposure prophylaxis.

^b^TFVdp: tenofovir diphosphate.

^c^IPV: intimate partner violence.

^d^WHO VAWI: World Health Organization’s Violence Against Women instrument.

^e^FTCtp: emtricitabine triphosphate.

The secondary outcomes of the study will be the proportion of participants reporting any IPV and severe IPV (Table 4) between enrollment and month 6, and between month 6 and month 12, and the difference in IPV intensity scores at months 6 and 12. The aim 2 hypothesis is that the proportion of participants reporting any IPV and severe IPV and IPV severity scores will all be *lower* at both time points in the intervention arm versus control arm.

In exploratory analyses, we will first explore effect modification by age (15-19 vs 20-24) and DREAMS participation (current, former, or never) for the primary and secondary outcomes. Second, we will break down the primary outcome into steps along the continuum (PrEP uptake, execution, and persistence) and IPV outcomes by type—physical, sexual, emotional, and economic—to understand where the intervention had its strongest effects. Third, we will compare the proportion of participants per arm with emtricitabine triphosphate (FTCtp) levels indicating consistent PrEP adherence over the past 2 weeks. Comparing TFVdp and FTCtp levels will yield insights on adherence patterns over both a long and short window of time before collection. We will also analyze the alignment of adherence measures with HIV risk perception and behavior to better understand adherence patterns.

#### Analysis Approach

Descriptive and bivariate statistics will be used to compare participant characteristics in the intervention and control arms and those who completed the study versus those lost to follow-up. Outcomes will be modeled using an intent-to-treat approach with mixed effect logistic and linear regression models. Model predictors will include treatment assignments, randomization strata, and a random effect for ward (cluster) within stratum that is assumed to be independent and normally distributed with mean 0. If the cluster randomization does not adequately balance baseline characteristics across arms, we will conduct exploratory analyses controlling for those characteristics. We will analyze the data from each of the 2 time points separately and the primary outcomes analyses will use a type I error rate of 0.025 at each time point.

#### Missing Data

In any situation with missing data, we will perform appropriate secondary analyses adjusting for variables that may be related to the missingness mechanism. If missing data rates are higher than anticipated (>10%), we will include covariates that are related to missingness in regression models. We will also perform sensitivity analyses to assess the potential impact of the missing data.

### Data Analysis: Process Evaluation

#### Quantitative Data

Implementation inputs, processes, and outputs will be analyzed descriptively, and we will draw comparisons across intervention wards. Intervention effects on each mechanism of change will be modeled individually following the approach described for the cRCT. We will regress each mechanism on the exposure (intervention arm assignment), controlling for baseline levels of the mechanism. If the primary analyses show no effect of the intervention on PrEP or IPV outcomes, the results will inform our interpretation of whether the intervention failed to affect the intended mechanism or whether the change in mechanism did not lead to the hypothesized outcome. If the primary analyses suggest a positive intervention effect on PrEP or IPV outcomes, we will conduct causal mediation analysis [72,73]. Coverage, uptake, and exit survey data on perceived impact will also be evaluated as moderators of intervention effectiveness at the site level in exploratory analyses of mechanisms of change.

#### Qualitative Data

The coding process will involve a core group of about 2 to 5 analysts from IRDO and RTI who will develop one or more codebooks and establish qualitative coding procedures. Data will be coded using a qualitative software package, such as Dedoose (SocioCultural Research Consultants, LLC). A set of preliminary codes will be developed based on key themes or topics identified a priori and the findings highlighted in the debriefing reports for each of the 3 datasets (eg, adolescent girls and young women, male partners, and providers). Each group member will apply the initial set of thematic codes to a common transcript, discuss their coding experiences, and agree on expanding and modifying code names and definitions when necessary. We will continue with an iterative process of revising the codebooks as we continue to read and code the data. Once finalized, the codebooks will be used for a final recoding of all transcripts.

A preselected number of transcripts will be double-coded by 2 coders to establish intracoder and intercoder reliability. Following this process, the coding team will discuss discrepancies, which will ultimately be resolved through consensus. This process will continue until the intercoder reliability is at least 80%. Thereafter the remaining texts will primarily be coded by 1 analyst only, with one or more rounds of consistency checks and regular discussions among the coding team to ensure that coding remains standardized and reliable.

Comprehensive listings of all coded quotations for every code or code “family” will be generated in the qualitative analysis software. Coding memos will be used to summarize and explore the relationship between the constructs of the evaluation framework (eg, the relationship between implementation processes and hypothesized mechanisms of change, or between mechanisms of change and outcomes). Analysts will also review and analyze coded serial IDI content within each participant’s dataset to look specifically for patterns over time. The findings and interpretations of the data will be critically discussed until there is group consensus on the dominant themes and meanings contained in the data [74].

#### Integrated Synthesis of Quantitative and Qualitative Data

For each construct of the evaluation framework, findings from the qualitative and quantitative analyses will be integrated to build a more comprehensive picture of whether the target intervention inputs, processes, and outputs were achieved, whether these resulted in the hypothesized changes in mechanisms of action, whether these changes resulted in the intended outcomes, and how external contextual factors influenced implementation, thereby both evaluating the success of study implementation and testing the theory of change.

### Study Monitoring

#### Protocol Oversight

The study team continuously reviews recruitment and follow-up data to ensure compliance with the protocol and ethical standards. The IRDO study coordinator regularly monitors protocol compliance at study sites. The IRDO internal research monitor conducts quarterly spot checks of 100% of informed consent forms and 10% of data files. Any issues are documented in quality control logs and reviewed with the study staff. Protocol deviations or violations are reported to the study leadership and institutional review board (IRB) within 5 business days.

#### Data and Safety Monitoring Board

The trial is monitored by a data and safety monitoring board (DSMB) composed of 4 members: a statistician, a medical physician, a researcher with content expertise in IPV, and a researcher with expertise in PrEP use among adolescent girls and young women. All DSMB members are independent from any professional or financial conflict of interest with the research project, study investigators, and study institutions. The DSMB established a charter that outlines their responsibilities and meets approximately semiannually to review data quality and integrity, protocol adherence, participant safety, study conduct, and progress. On the basis of their review, they make recommendations on study continuation and modifications.

#### Safety Monitoring

This study adheres to the World Health Organization Ethical and Safety Recommendations for Intervention Research on Violence Against Women [75]. All data collection staff receive training on IPV assessment, recognizing warning signs of immediate danger, discussing sensitive topics with participants, managing distressed participants, legal reporting requirements, and referral protocols. We aim to ensure that all communication with participants protects against unintentional disclosure of PrEP use, study participation, or reported IPV and does not place participants at additional risk of harm. To increase confidentiality, the study is presented to the community and to the participants as a study on HIV prevention methods in young women, and the focus on IPV is not advertised.

The MoH clinics conduct clinical safety monitoring for PrEP according to Kenyan guidelines. This study focuses on monitoring for adverse events that could be associated with the intervention, including IPV, social harms, serious adverse events, and unanticipated problems. All instances of these events are recorded on case report forms and followed up until resolution or 30 days after the participant exits from the study. These adverse events are reviewed monthly by the protocol team and reported to the IRB and DSMB according to their established protocols.

All staff have been trained to respond to any reports on IPV and social harms using the World Health Organization’s Listen, Inquire, Validate, Enhance Safety, and Support (LIVES) approach through referrals [76]. Staff follow up by phone or during subsequent visits to check on referral uptake and provide additional assistance if needed. Participants who report being in immediate danger or feeling unsafe returning home are offered an immediate escorted referral to vetted rescue or shelter services, and study staff follow up with the participant weekly until their situation is resolved or stabilized, or until 30 days after their exit from the study. If staff have concerns about the safety of a participant’s enrollment, they alert the site PI or designee to make a final determination about eligibility.

### Ethical Considerations

The trial is being carried out in accordance with International Council on Harmonization Good Clinical Practice and the United States Code of Federal Regulations applicable to clinical studies (45 CFR part 46).

#### Ethics Approval

The study protocol and associated documents have been reviewed and approved by the Maseno University Ethical Review Committee (MUERC; MSU/DRPI/MUERC/00991/21). The RTI International Committee for Human Subjects Research established a reliance on MUERC for the study under a signed IRB authorization agreement. No amendment to the protocol or informed consent forms is implemented without prior ethics committee approval. Protocol violations are reported in writing to MUERC in accordance with their policy.

#### Informed Consent Process

IRDO study staff obtain informed consent or assent from all participants taking part in the cRCT or IDIs. Study staff explain the study purpose and procedures, risks and benefits to the eligible participants, and compensation for their participation. They emphasize that their participation is voluntary and that there is no penalty if the individual decides not to participate. Participants aged ≥18 years provide written consent in English, Kiswahili, or Dholuo. The IRB has granted a waiver of parental consent for adolescent girls and young women participants who meet the criteria for “mature minors” outlined in MoH guidelines for Conducting Adolescent HIV Sexual and Reproductive Health Research in Kenya [77], and for male partners aged 15 to 17 years to take part in IDIs, because they constitute a minimal-risk research activity. All minor participants may choose to involve their parent in the consent process. For minor participants not meeting the above criteria, or who involve their parents, we obtain parental consent and child assent. The IRB has also granted a waiver of written consent for the brief and anonymous exit interviews focused on program acceptability.

#### Confidentiality

All study procedures occur in private settings. Each participant receives a unique study identification number, and personally identifiable information does not appear on study data. All electronic data and study records are stored on secure, password-protected devices and uploaded to a secure RTI project server. Access to the system is restricted to trained study staff. Hard-copy data, consent and contact information forms with personally identifiable information are kept in separate double-locked files at the site and not transmitted to RTI.

To minimize confidentiality risks during the follow-up visits, we use contact methods agreed to by the participants, including aliases for study staff. To protect confidentiality during group activities, participants are asked to follow ground rules and pledge not to disclose others’ personal details outside the group. At each session’s start and end, participants are reminded to respect confidentiality and that there is a risk that others may disclose what they say.

Participants’ study information will not be released without their written permission, except as required by law or as necessary for review, monitoring, and auditing by the following:

1. Representatives of the US federal government, including the United States Office of Human Research Protections, the National Institutes of Health (NIH) and contractors of the NIH, and other local, United States, and international regulatory entities
2. Representatives of RTI
3. Study staff
4. Site IRBs

#### Criteria for Early Termination of Study Participation

Participants can withdraw from the study at any time for any reason. The PI, site PI, or designee may also withdraw participants for safety or noncompliance reasons, with the DSMB notified of such terminations. Efforts are made to complete a final evaluation for participants who withdraw. Staff document withdrawal reasons in study records. Participants who withdraw may rejoin and resume intervention or follow-up visits up to their original exit date, with PI and DSMB consultation.

#### Study Discontinuation

The study may be suspended or terminated at any time by the NIH, RTI, site IRBs, or other authorities due to funding issues or substantial concerns, such as DSMB recommendations. If this occurs, the PI will be notified in writing with the reasons, and will inform participants, the IRB, and the sponsor. The study may resume if safety, protocol compliance, and data quality issues are resolved and approved by the relevant parties.

### Dissemination and Use of Findings

Study findings will be disseminated to the study communities, the scientific community, and HIV service and advocacy organizations via peer review publications, professional meetings, research briefs, and stakeholder meetings. All media, presentations, and publications resulting from data collected during this study will be collaborative in nature but require approval from the study leadership. All publications will be submitted to PubMed Central, in accordance with NIH Public Access Policy.

## Results

The study was funded in August 2021, and data collection started in September 2022. As of December 2024, enrollment has been completed in 16 of the 22 study wards, with 72.6% (1150/1584) participants enrolled. We anticipate that data collection will be completed in May 2026 and results will be available by mid-2027.

## Discussion

### Innovations and Future Application

This study offers 4 key innovations. First, it offers the opportunity to address the lack of evidence-based interventions that explicitly confront the role of IPV and relationship dynamics in supporting PrEP use among adolescent girls and young women. Current interventions have focused largely on instrumental adherence support (eg, pill-taking reminders and strategies), motivational interviewing, and peer support, with limited evidence of success [4,5,78-83]. If found effective, the Tu’Washindi intervention will be one of the first to successfully address critical partner-related barriers to uptake and adherence and to directly involve male partners to build their support for PrEP use among adolescent girls and young women. Second, our intervention approach is youth-designed and tailored to meet the specific needs of adolescent girls and young women, qualities that have been specifically called for in recent literature to address shortcomings in existing interventions [84,85]. The intervention components of male engagement and couples’ education were added to the design in direct response to the formative research findings, and our participatory design methods resulted in high intervention acceptability and uptake among adolescent girls and young women and high ratings for relevance and perceived effectiveness. Third, our multilevel approach recognizes that IPV and relationship power create barriers to HIV prevention at the individual, partnership, and community levels. Tu’Washindi supports adolescent girls and young women to respond to experienced or anticipated IPV at each level by fostering self-efficacy, improving relationship communication, developing support systems, and encouraging action to maintain safety and HIV prevention adherence [86-88]. Fourth, we assess short- and long-term adherence with biomarker measurements of 2 active PrEP metabolites, TFVdp and FTCtp to gain a more thorough understanding of adolescent girls and young women adherence behavior. Although the field has focused on TFVdp levels to classify long-term (ie, previous 1-3 months) PrEP adherence [89], FTCtp concentrations can delineate between consistent (≥4 doses/week) versus inconsistent (<4 doses/week) PrEP adherence over 2 weeks before sample collection with ≥89% accuracy [90]. Thus, we will have the ability to classify adolescent girls and young women by short-term (2 weeks) adherence in addition to the long-term (2 months) adherence measured by TFVdp levels.

Our intervention and evaluation have been designed from the start to facilitate scalability, sustainability, and rapid adoption. Tu’Washindi was designed to be delivered by trained community members with support from clinicians and counselors, and to integrate into ongoing national or donor-funded youth-focused programs prioritizing HIV prevention. The proposed study uses a pragmatic, rather than exploratory, design to maximize generalizability and ensure results are applicable to real-world settings [44,91]. In addition, our design includes a rigorous process evaluation to document implementation process, quality and fidelity of delivery, resource requirements, and contextual factors to provide valuable guidance to program and policy stakeholders who wish to replicate or adapt the intervention outside of the research setting. In combination, the intervention design and process evaluation approach will reduce the “know-do gap” [92] and accelerate Tu’Washindi’s translation from research to practice.

### Limitations

Several limitations to this study should be noted. First, there is a risk of PrEP and HIV test kit shortages in MoH clinics, which could interfere with adherence and complicate interpretation of results. We will carefully document shortages as part of the program diary, and study staff will help link participants to other sources of PrEP provision if necessary. Second, cluster randomized studies carry a risk that individual participant characteristics may differ at baseline in important ways. Our design stratifies on 2 important community characteristics (ie, geographic type and baseline PrEP use) to reduce this risk, and our analysis plan incorporates exploratory analyses to control for baseline differences between randomization arms.

### Conclusions

Tu’Washindi has demonstrated feasibility and acceptability in pilot research and shows promise to promote PrEP use and reduce IPV among adolescent girls and young women. The proposed study builds directly on our intervention development work to conduct a rigorous effectiveness trial, a critical step in developing the evidence base for this youth-designed, multilevel HIV prevention intervention. Our rigorous process evaluation will clarify mechanisms of change, and implementation considerations to inform policy and practice. If effective, Tu’Washindi will be ideally positioned for sustainable integration into existing youth-focused HIV prevention programming to expand and support PrEP uptake and adherence in this priority population.

## Appendix

Multimedia Appendix 1 SPIRIT (Standard Protocol Items: Recommendations for Interventional Trials) 2013 checklist: recommended items to address in a clinical trial protocol and related documents.

Multimedia Appendix 2 Peer-review report by the Center for Scientific Review HIV/AIDS Intra- and Inter-personal Determinants and Behavioral Interventions Study Section (National Institutes of Health, USA).

## Abbreviations

cRCT: cluster randomized controlled trial
DBS: dried blood spot
DREAMS: Determined, Resilient, Empowered, AIDS-free, Mentored and Safe
DSMB: data and safety monitoring board
FTCtp: emtricitabine triphosphate
IDI: in-depth interview
IPV: intimate partner violence
IRB: institutional review board
IRDO: Impact Research and Development Organization
LIVES: Listen, Inquire, Validate, Enhance Safety, and Support
MoH: Ministry of Health
MUERC: Maseno University Ethical Review Committee
NIH: National Institutes of Health
PI: principal investigator
PrEP: preexposure prophylaxis
REDCap: Research Electronic Data Capture
TFVdp: tenofovir diphosphate

## Data Availability

The research tools, protocols, standard operating procedures, analytic code, codebooks, and deidentified data generated in this study will be available from the corresponding author upon reasonable request. Before access to deidentified participant data is granted, a data sharing agreement will be implemented to ensure that the data will be used for the proposed purpose and that no attempts will be made to identify participants.

## References

[1] (2019). Communities at the centre: defending rights, breaking barriers, reaching people with HIV services. Joint United Nations Programme on HIV/AIDS.

[2] We've got the power: women, adolescent girls, and the HIV response. Joint United Nations Programme on HIV/AIDS.

[3] Blaschke TF, Osterberg L, Vrijens B, Urquhart J (2012). Adherence to medications: insights arising from studies on the unreliable link between prescribed and actual drug dosing histories. Annu Rev Pharmacol Toxicol.

[4] Celum C, Gill K, Morton J, Stein G, Myers L, Thomas K, McConnell M, van der Straten A, Baeten JM, Duyver M, Mendel E, Naidoo K, Dallimore J, Wiesner L, Bekker LG (2020). Incentives conditioned on tenofovir levels to support PrEP adherence among young South African women: a randomized trial. J Int AIDS Soc.

[5] Celum C, Hosek S, Tsholwana M, Kassim S, Mukaka S, Dye BJ, Pathak S, Mgodi N, Bekker LG, Donnell DJ, Wilson E, Yuha K, Anderson PL, Agyei Y, Noble H, Rose SM, Baeten JM, Fogel JM, Adeyeye A, Wiesner L, Rooney J, Delany-Moretlwe S (2021). PrEP uptake, persistence, adherence, and effect of retrospective drug level feedback on PrEP adherence among young women in southern Africa: Results from HPTN 082, a randomized controlled trial. PLoS Med.

[6] Tapsoba JD, Cover J, Obong'o C, Brady M, Cressey TR, Mori K, Okomo G, Kariithi E, Obanda R, Oluoch-Madiang D, Chen YQ, Drain P, Duerr A (2022). Continued attendance in a PrEP program despite low adherence and non-protective drug levels among adolescent girls and young women in Kenya: results from a prospective cohort study. PLoS Med.

[7] de Dieu Tapsoba J, Zangeneh SZ, Appelmans E, Pasalar S, Mori K, Peng L, Tao J, Drain P, Okomo G, Bii S, Mukabi J, Zobrist S, Brady M, Obanda R, Madiang DO, Cover J, Duerr A, Chen YQ, Obong'o C (2021). Persistence of oral pre-exposure prophylaxis (PrEP) among adolescent girls and young women initiating PrEP for HIV prevention in Kenya. AIDS Care.

[8] Gill K, Johnson L, Dietrich J, Myer L, Marcus R, Wallace M, Pidwell T, Mendel E, Fynn L, Jones K, Wiesner L, Slack C, Strode A, Spiegel H, Hosek S, Rooney J, Gray G, Bekker L (2020). Acceptability, safety, and patterns of use of oral tenofovir disoproxil fumarate and emtricitabine for HIV pre-exposure prophylaxis in South African adolescents: an open-label single-arm phase 2 trial. Lancet Child Adolesc Health.

[9] Haberer JE, Bangsberg DR, Baeten JM, Curran K, Koechlin F, Amico KR, Anderson P, Mugo N, Venter F, Goicochea P, Caceres C, O'Reilly K (2015). Defining success with HIV pre-exposure prophylaxis: a prevention-effective adherence paradigm. AIDS.

[10] Kinuthia J, Pintye J, Abuna F, Mugwanya KK, Lagat H, Onyango D, Begnel E, Dettinger J, Baeten JM, John-Stewart G, PrEP Implementation for Young Women and Adolescents (PrIYA) programme (2020). Pre-exposure prophylaxis uptake and early continuation among pregnant and post-partum women within maternal and child health clinics in Kenya: results from an implementation programme. Lancet HIV.

[11] Kyongo J, Kiragu M, Karuga R, Ochieng C, Ngunjiri A, Wachihi C, Musyoki H, Digolo L, Otiso L, Gelmon L, Kilonzo N, Mukoma W (2018). How long will they take it? Oral pre-exposure prophylaxis (PrEP) retention for female sex workers, men who have sex with men and young women in a demonstration project in Kenya. J Int AIDS Soc.

[12] Masyuko S, Mukui I, Njathi O, Kimani M, Oluoch P, Wamicwe J, Mutegi J, Njogo S, Anyona M, Muchiri P, Maikweki L, Musyoki H, Bahati P, Kyongo J, Marwa T, Irungu E, Kiragu M, Kioko U, Ogando J, Were D, Bartilol K, Sirengo M, Mugo N, Baeten JM, Cherutich P, PrEP Technical Working Group (2018). Pre-exposure prophylaxis rollout in a national public sector program: the Kenyan case study. Sex Health.

[13] Mukui I (2018). Understanding PrEP effectiveness in different populations in the context of public health programs. Proceedings of the 2018 International Conference on HIV Research for Prevention.

[14] Oluoch LM, Roxby A, Mugo N, Wald A, Ngure K, Selke S, Chohan B, Kiptinness C, Tapia K, Micheni M, Maina SG, Casmir E (2021). Does providing laboratory confirmed STI results impact uptake of HIV pre-exposure prophylaxis (PrEP) uptake among Kenyan adolescents girls and young women? A descriptive analysis. Sex Transm Infect.

[15] Ndimande-Khoza MN, Katz AW, Moretlwe-Delany S, Travill D, Rousseau E, Omollo V, Morton J, Johnson R, Bekker L, Bukusi EA, Baeten J, Celum C, van der Straten A, Roberts ST (2023). Family influences on oral PrEP use among adolescent girls and young women in Kenya and South Africa. PLoS One.

[16] Sila J, Larsen AM, Kinuthia J, Owiti G, Abuna F, Kohler PK, John-Stewart G, Pintye J (2020). High awareness, yet low uptake, of pre-exposure prophylaxis among adolescent girls and young women within family planning clinics in Kenya. AIDS Patient Care STDS.

[17] Were D, Musau A, Mutegi J, Ongwen P, Manguro G, Kamau M, Marwa T, Gwaro H, Mukui I, Plotkin M, Reed J (2020). Using a HIV prevention cascade for identifying missed opportunities in PrEP delivery in Kenya: results from a programmatic surveillance study. J Int AIDS Soc.

[18] Kenya National Bureau of Statistics, Ministry of Health/Kenya, National AIDS Control Council/Kenya, Kenya Medical Research Institute, National Council for Population and Development/Kenya (2015). Kenya Demographic and Health Survey 2014.

[19] Mathur S, Okal J, Musheke M, Pilgrim N, Kishor Patel S, Bhattacharya R, Jani N, Matheka J, Banda L, Mulenga D, Pulerwitz J (2018). High rates of sexual violence by both intimate and non-intimate partners experienced by adolescent girls and young women in Kenya and Zambia: findings around violence and other negative health outcomes. PLoS One.

[20] Pulerwitz J, Mathur S, Woznica D (2018). How empowered are girls/young women in their sexual relationships? Relationship power, HIV risk, and partner violence in Kenya. PLoS One.

[21] Jewkes RK, Dunkle K, Nduna M, Shai N (2010). Intimate partner violence, relationship power inequity, and incidence of HIV infection in young women in South Africa: a cohort study. Lancet.

[22] Kouyoumdjian FG, Calzavara LM, Bondy SJ, O'Campo P, Serwadda D, Nalugoda F, Kagaayi J, Kigozi G, Wawer M, Gray R (2013). Intimate partner violence is associated with incident HIV infection in women in Uganda. AIDS.

[23] Li Y, Marshall CM, Rees HC, Nunez A, Ezeanolue EE, Ehiri JE (2014). Intimate partner violence and HIV infection among women: a systematic review and meta-analysis. J Int AIDS Soc.

[24] Allsworth JE, Secura GM, Zhao Q, Madden T, Peipert JF (2013). The impact of emotional, physical, and sexual abuse on contraceptive method selection and discontinuation. Am J Public Health.

[25] Bergmann JN, Stockman JK (2015). How does intimate partner violence affect condom and oral contraceptive use in the United States?: a systematic review of the literature. Contraception.

[26] Hampanda KM (2016). Intimate partner violence and HIV-positive women's non-adherence to antiretroviral medication for the purpose of prevention of mother-to-child transmission in Lusaka, Zambia. Soc Sci Med.

[27] Maxwell L, Devries K, Zionts D, Alhusen JL, Campbell J (2015). Estimating the effect of intimate partner violence on women's use of contraception: a systematic review and meta-analysis. PLoS One.

[28] Sprague C, Hatcher AM, Woollett N, Black V (2017). How nurses in Johannesburg address intimate partner violence in female patients: understanding IPV responses in low- and middle-income country health systems. J Interpers Violence.

[29] Stephenson R, Bartel D, Rubardt M (2012). Constructs of power and equity and their association with contraceptive use among men and women in rural Ethiopia and Kenya. Glob Public Health.

[30] Brown JL, Young AM, Sales JM, DiClemente RJ, Rose ES, Wingood GM (2014). Impact of abuse history on adolescent African-American women's current HIV/STD-associated behaviors and psychosocial mediators of HIV/STD risk. J Aggress Maltreat Trauma.

[31] Hatcher AM, Stöckl H, Christofides N, Woollett N, Pallitto CC, Garcia-Moreno C, Turan JM (2016). Mechanisms linking intimate partner violence and prevention of mother-to-child transmission of HIV: a qualitative study in South Africa. Soc Sci Med.

[32] Salazar LF, Crosby RA, DiClemente RJ, Wingood GM, Lescano CM, Brown LK, Harrington K, Davies S (2005). Self-esteem and theoretical mediators of safer sex among African American female adolescents: implications for sexual risk reduction interventions. Health Educ Behav.

[33] Lanham M, Wilcher R, Montgomery ET, Pool R, Schuler S, Lenzi R, Friedland B (2014). Engaging male partners in women's microbicide use: evidence from clinical trials and implications for future research and microbicide introduction. J Int AIDS Soc.

[34] Montgomery ET, van der Straten A, Stadler J, Hartmann M, Magazi B, Mathebula F, Laborde N, Soto-Torres L (2015). Male partner influence on women's HIV prevention trial participation and use of pre-exposure prophylaxis: the importance of "understanding". AIDS Behav.

[35] Stadler J, Delany-Moretlwe S, Palanee T, Rees H (2014). Hidden harms: women's narratives of intimate partner violence in a microbicide trial, South Africa. Soc Sci Med.

[36] Hartmann M, Otticha S, Agot K, Wanga B, Oginga F, Minnis A (2019). Unpacking the role of gender-based violence as a barrier to pre-exposure prophylaxis use among adolescent girls and young women in the DREAMS program in Kenya through qualitative storytelling. Proceedings of the 2019 International Conference on International Workshop on HIV & Adolescence.

[37] van der Straten A, Stadler J, Luecke E, Laborde N, Hartmann M, Montgomery ET, VOICE-C Study Team (2014). Perspectives on use of oral and vaginal antiretrovirals for HIV prevention: the VOICE-C qualitative study in Johannesburg, South Africa. J Int AIDS Soc.

[38] van der Straten A, Stadler J, Montgomery E, Hartmann M, Magazi B, Mathebula F, Schwartz K, Laborde N, Soto-Torres L (2014). Women's experiences with oral and vaginal pre-exposure prophylaxis: the VOICE-C qualitative study in Johannesburg, South Africa. PLoS One.

[39] Velloza J, Khoza N, Scorgie F, Chitukuta M, Mutero P, Mutiti K, Mangxilana N, Nobula L, Bulterys MA, Atujuna M, Hosek S, Heffron R, Bekker L, Mgodi N, Chirenje M, Celum C, Delany-Moretlwe S, HPTN 082 study group (2020). The influence of HIV-related stigma on PrEP disclosure and adherence among adolescent girls and young women in HPTN 082: a qualitative study. J Int AIDS Soc.

[40] Hartmann M, Otticha S, Agot K, Minnis AM, Montgomery ET, Roberts ST (2021). Tu'Washindi na PrEP: working with young women and service providers to design an intervention for PrEP uptake and adherence in the context of gender-based violence. AIDS Educ Prev.

[41] Otticha S, Hartmann M, Agot K, Minnis A, Roberts ST (2020). Working with young people to design and implement an intervention for PrEP uptake and persistence. Proceedings of the 23rd International AIDS Conference.

[42] Minnis AM, Agot K, Hartmann M, Otticha S, Montgomery ET, Roberts ST (2024). Feasibility and acceptability of the novel Tu'Washindi intervention to increase PrEP use among adolescent girls and young women in Siaya county, Kenya. AIDS Behav.

[43] Roberts ST, Hartmann M, Minnis AM, Otticha SO, Browne EN, Montgomery ET, Agot K (2023). Breaking down relationship barriers to increase PrEP uptake and adherence among adolescent girls and young women in Kenya: safety and preliminary effectiveness results from a pilot cluster-randomized trial. J Int AIDS Soc.

[44] Treweek S, Zwarenstein M (2009). Making trials matter: pragmatic and explanatory trials and the problem of applicability. Trials.

[45] (2015). County Government of Siaya. Republic of Kenya.

[46] Kenya HIV prevention revolution road map. Ministry of Health, Kenya.

[47] Ohiomoba RO, Owuor PM, Orero W, Were I, Sawo F, Ezema A, Jackson-Gibson M, Hirschhorn LR (2022). Pre-exposure prophylaxis (PrEP) initiation and retention among young Kenyan women. AIDS Behav.

[48] (2024). Translating progress into success: a decade of progress report, 2013-2023. Kenya National Syndemic Diseases Control Council.

[49] Bandura A, DiClemente RJ, Peterson JL (1994). Social cognitive theory and exercise of control over HIV infection. Preventing AIDS: Theories and Methods of Behavioral Interventions.

[50] Mathur S, Pilgrim N, Pulerwitz J (2016). PrEP introduction for adolescent girls and young women. Lancet HIV.

[51] Pettifor A, Lippman SA, Selin AM, Peacock D, Gottert A, Maman S, Rebombo D, Suchindran CM, Twine R, Lancaster K, Daniel T, Gómez-Olivé FX, Kahn K, MacPhail C (2015). A cluster randomized-controlled trial of a community mobilization intervention to change gender norms and reduce HIV risk in rural South Africa: study design and intervention. BMC Public Health.

[52] (2015). Kenya demographic and health survey. Kenya Bureau of National Statistics.

[53] Agot K, Hartmann M, Otticha S, Minnis A, Onyango J, Ochillo M, Roberts ST (2022). " I didn't support PrEP because I didn't know what it was": inadequate information undermines male partner support for young women's pre-exposure prophylaxis use in western Kenya. Afr J AIDS Res.

[54] Doyle AM, Floyd S, Baisley K, Orindi B, Kwaro D, Mthiyane TN, Muuo S, Shahmanesh M, Ziraba A, Birdthistle I (2018). Who are the male sexual partners of adolescent girls and young women? Comparative analysis of population data in three settings prior to DREAMS roll-out. PLoS One.

[55] Theatre facilitation manual. Health Communication Partnership, Zambia.

[56] Saul J, Bachman G, Allen S, Toiv NF, Cooney C, Beamon TA (2018). The DREAMS core package of interventions: a comprehensive approach to preventing HIV among adolescent girls and young women. PLoS One.

[57] (2018). Guidelines on use of antiretroviral drugs for treating and preventing HIV in Kenya, 2018. Ministry of Health, National AIDS & STI Control Program.

[58] Freedland KE, King AC, Ambrosius WT, Mayo-Wilson E, Mohr DC, Czajkowski S, Thabane L, Collins LM, Rebok GW, Treweek SP, Cook TD, Edinger JD, Stoney CM, Campo RA, Young-Hyman D, Riley WT, National Institutes of Health Office of Behavioral and Social Sciences Research Expert Panel on Comparator Selection in Behavioral and Social Science Clinical Trials (2019). The selection of comparators for randomized controlled trials of health-related behavioral interventions: recommendations of an NIH expert panel. J Clin Epidemiol.

[59] (2019). The United States President’s Emergency Plan for AIDS Relief 2019: annual report to Congress. U.S. Department of State Office of the U.S.

[60] Schauer AP, Sykes C, Cottrell ML, Prince H, Kashuba AD (2018). Validation of an LC-MS/MS assay to simultaneously monitor the intracellular active metabolites of tenofovir, emtricitabine, and lamivudine in dried blood spots. J Pharm Biomed Anal.

[61] Moore GF, Audrey S, Barker M, Bond L, Bonell C, Hardeman W, Moore L, O'Cathain A, Tinati T, Wight D, Baird J (2015). Process evaluation of complex interventions: medical research council guidance. BMJ.

[62] Plummer ML, Wight D, Obasi AI, Wamoyi J, Mshana G, Todd J, Mazige BC, Makokha M, Hayes RJ, Ross DA (2007). A process evaluation of a school-based adolescent sexual health intervention in rural Tanzania: the MEMA kwa Vijana programme. Health Educ Res.

[63] Power R, Langhaug LF, Nyamurera T, Wilson D, Bassett MT, Cowan FM (2004). Developing complex interventions for rigorous evaluation--a case study from rural Zimbabwe. Health Educ Res.

[64] Busza J, Chiyaka T, Musemburi S, Fearon E, Davey C, Chabata S, Mushati P, Dirawo J, Napierala S, Phillips AN, Cowan FM, Hargreaves JR (2019). Enhancing national prevention and treatment services for sex workers in Zimbabwe: a process evaluation of the SAPPH-IRe trial. Health Policy Plan.

[65] Hatcher AM, McBride RS, Rebombo D, Munshi S, Khumalo M, Christofides N (2020). Process evaluation of a community mobilization intervention for preventing men's partner violence use in peri-urban South Africa. Eval Program Plann.

[66] Harris PA, Taylor R, Minor BL, Elliott V, Fernandez M, O'Neal L, McLeod L, Delacqua G, Delacqua F, Kirby J, Duda SN, REDCap Consortium (2019). The REDCap consortium: building an international community of software platform partners. J Biomed Inform.

[67] Harris PA, Taylor R, Thielke R, Payne J, Gonzalez N, Conde JG (2009). Research electronic data capture (REDCap)--a metadata-driven methodology and workflow process for providing translational research informatics support. J Biomed Inform.

[68] Devanathan AS, Dumond JB, Anderson DJ, Moody K, Poliseno AJ, Schauer AP, Sykes C, Gay CL, Rosen EP, Kashuba AD, Cottrell ML (2023). A novel algorithm to improve PrEP adherence monitoring using dried blood spots. Clin Pharmacol Ther.

[69] Anderson PL, Glidden DV, Liu A, Buchbinder S, Lama JR, Guanira JV, McMahan V, Bushman LR, Casapía M, Montoya-Herrera O, Veloso VG, Mayer KH, Chariyalertsak S, Schechter M, Bekker L, Kallás EG, Grant RM, iPrEx Study Team (2012). Emtricitabine-tenofovir concentrations and pre-exposure prophylaxis efficacy in men who have sex with men. Sci Transl Med.

[70] Garcia-Moreno C, Jansen HA, Ellsberg M, Heise L, Watts CH, WHO Multi-country Study on Women's Health and Domestic Violence against Women Study Team (2006). Prevalence of intimate partner violence: findings from the WHO multi-country study on women's health and domestic violence. Lancet.

[71] Heise A, Hossain M (2017). STRIVE technical brief: measuring intimate partner violence. London School of Hygeine and Tropical Medicine.

[72] VanderWeele T, Vansteelandt S (2009). Conceptual issues concerning mediation, interventions and composition. Stat Interface.

[73] Valeri L, Vanderweele TJ (2013). Mediation analysis allowing for exposure-mediator interactions and causal interpretation: theoretical assumptions and implementation with SAS and SPSS macros. Psychol Methods.

[74] Ryan GW, Bernard RH, Denzin N, Lincoln Y (2000). Data management and analysis methods. Handbook of Qualitative Research. 2nd edition.

[75] (2016). Ethical and safety recommendations for intervention research on violence against women. Building on lessons from the WHO publication Putting women first: ethical and safety recommendations for research on violence against women. World Heatlh Organization.

[76] (2014). Health care for women subjected to intimate partner violence or sexual violence: a clinical handbook. World Health Organization.

[77] National AIDS and STI Control Program (NASCOP), Kenya Medical Research Institiute (KEMRI) (2015). Guidelines for conducting adolescents sexual and reproductive health research in Kenya. Government of Kenya Ministry of Health.

[78] Amico KR, Mansoor LE, Corneli A, Torjesen K, van der Straten A (2013). Adherence support approaches in biomedical HIV prevention trials: experiences, insights and future directions from four multisite prevention trials. AIDS Behav.

[79] Amico KR, Mugavero M, Krousel-Wood MA, Bosworth HB, Merlin JS (2018). Advantages to using social-behavioral models of medication adherence in research and practice. J Gen Intern Med.

[80] Amico KR, Wallace M, Bekker LG, Roux S, Atujuna M, Sebastian E, Dye BJ, Elharrar V, Grant RM (2017). Experiences with HPTN 067/ADAPT study-provided open-label PrEP among women in Cape Town: facilitators and barriers within a mutuality framework. AIDS Behav.

[81] Amico RK, McMahan V, Goicochea P, Vargas L, Marcus JL, Grant RM, Liu A (2012). Supporting study product use and accuracy in self-report in the iPrEx study: next step counseling and neutral assessment. AIDS Behav.

[82] Balán IC, Lentz C, Giguere R, Mayo AJ, Rael CT, Soto-Torres L, Palanee-Phillips T, Mgodi NM, Hillier S, Baeten JM, MTN-025/HOPE Study Team (2020). Implementation of a fidelity monitoring process to assess delivery of an evidence-based adherence counseling intervention in a multi-site biomedical HIV prevention study. AIDS Care.

[83] Delany-Moretlwe S, Chersich M, Scorgie F, Baron D, Harvey S, Colombini M, Naicker N, Kapiga S, Stangl A, EMPOWER Study Team (2018). Empowerment clubs did not increase PrEP continuation among adolescent girls and young women in South Africa and Tanzania-Results from the EMPOWER randomised trial. J Int AIDS Soc.

[84] Mannell J, Willan S, Shahmanesh M, Seeley J, Sherr L, Gibbs A (2019). Why interventions to prevent intimate partner violence and HIV have failed young women in southern Africa. J Int AIDS Soc.

[85] Gibbs A (2016). Tackling gender inequalities and intimate partner violence in the response to HIV: moving towards effective interventions in Southern and Eastern Africa. Afr J AIDS Res.

[86] Campbell C, Mannell J (2016). Conceptualising the agency of highly marginalised women: intimate partner violence in extreme settings. Glob Public Health.

[87] Mannell J, Jackson S, Umutoni A (2016). Women's responses to intimate partner violence in Rwanda: rethinking agency in constrained social contexts. Glob Public Health.

[88] Logie CH, Daniel C (2016). 'My body is mine': qualitatively exploring agency among internally displaced women participants in a small-group intervention in Leogane, Haiti. Glob Public Health.

[89] Anderson PL, Liu AY, Castillo-Mancilla JR, Gardner EM, Seifert SM, McHugh C, Wagner T, Campbell K, Morrow M, Ibrahim M, Buchbinder S, Bushman LR, Kiser JJ, MaWhinney S (2018). Intracellular tenofovir-diphosphate and emtricitabine-triphosphate in dried blood spots following directly observed therapy. Antimicrob Agents Chemother.

[90] Anderson D, Prince H, Poliseno A, Moody K, Saunders A, Sykes C (2019). An exploration of adherence measures to detect recent changes in Truvada® dosing patterns. Proceedings of the 2019 International Workshop on Clinical Pharmacology on HIV, Hepatitis and Other Antiviral Drugs.

[91] Loudon K, Treweek S, Sullivan F, Donnan P, Thorpe KE, Zwarenstein M (2015). The PRECIS-2 tool: designing trials that are fit for purpose. BMJ.

[92] Lambdin BH, Cheng B, Peter T, Mbwambo J, Apollo T, Dunbar M, Udoh IC, Cattamanchi A, Geng EH, Volberding P (2015). Implementing implementation science: an approach for HIV prevention, care and treatment programs. Curr HIV Res.

