# Supplementary material for: Evaluation of the Tu’Washindi Na PrEP Intervention to Reduce Gender-Based Violence and Increase Preexposure Prophylaxis Uptake and Adherence Among Kenyan Adolescent Girls and Young Women: Protocol for a Cluster Randomized Controlled Trial
Source: JMIR Res Protoc. 2025 Apr 1;14:e55931. doi: 10.2196/55931 (PMC12000790; doi:10.2196/55931)
Supplement: Multimedia Appendix 2 [file resprot_v14i1e55931_app2.pdf]

1R01MH125671-01A1 Roberts, Sarah

EARLY STAGE INVESTIGATOR
NEW INVESTIGATOR

RESUME AND SUMMARY OF DISCUSSION: This application proposes to evaluate the effectiveness of a multilevel community-based intervention, developed by this team in a prior R34 application, to increase uptake and adherence to oral pre-exposure prophylaxis (PrEP) among adolescent girls and young women (AGYW) in Kenya. The intervention includes an eight-session, empowerment-based support club for AGYW, community sensitization targeted toward male partners, and PrEP education events for couples. The team will conduct a cluster-randomized controlled trial using 22 administrative wards in Siaya County, Kenya, to enroll 72 AGYW from each ward (total N=1,584) and randomly assign the participants from each ward to receive the intervention or usual HIV prevention care. The primary outcome of the proposed study will be PrEP uptake and adherence immediately post-delivery (month 6 post-enrollment) and 6 months later (month 12) using dried blood spots, and the secondary outcomes will examine the effect of the intervention on interpersonal violence (IPV) and relationship power. Finally, a process evaluation of the implementation will be conducted with the participants and the intervention providers. Given the low uptake of PrEP by this high risk group of AGYW in Kenya, the proposed intervention has the potential to have a significant public health impact. The study team is strong, and the expertise of the team has been improved by the addition of Kenyan pediatrician. The applicant has been highly responsive to the concerns raised in the prior review of this application. One substantial change was the separation of the study from the DREAMS initiative which should improve adolescent recruitment and, moreover, addressed concerns about the success of the intervention should the DREAMS initiative end. Concerns about the integration of input from the three advisory boards were also addressed. However, there were remaining concerns about the lack of evaluation of the male partners, the weak results of the pilot, and other minor methodological issues. These remaining areas of concerns served to limit the committee's overall enthusiasm for the potential impact for this otherwise much improved resubmission.

DESCRIPTION (provided by applicant): This R01 application, led by an Early Stage Investigator, aims to test the effectiveness of a multilevel community- based intervention to increase uptake and adherence to oral pre-exposure prophylaxis (PrEP) among adolescent girls and young women (AGYW) in Kenya. AGYW in this setting live in a context of heightened gender inequality and risk of intimate partner violence (IPV) and represent a large subpopulation who are uniquely vulnerable to HIV infection. In addition to having higher HIV incidence, women experiencing IPV are less likely to initiate and persist with PrEP, limiting the protective benefit of this efficacious biomedical prevention intervention. Barriers to PrEP uptake and adherence occur at multiple levels of the socioecological model, including low individual self-efficacy, partner opposition, and community stigma. Tu'Washindi na PrEP (We are Winners with PrEP) is a multi-level, community-based intervention designed specifically to address these challenges among AGYW in Siaya County, Kenya. Designed by our team using participatory methods engaging local AGYW (R34 MH114519), the intervention includes three components delivered over 6 months: an eight-session, empowerment-based support club for AGYW, community sensitization targeted toward male partners, and PrEP education events for couples. Activities are designed to be integrated into youth-focused programming to ensure efficiency and sustainability. Results from a pilot cluster- randomized controlled trial conducted with 100 AGYW at six sites indicate high feasibility and acceptability, and preliminary effectiveness: intervention arm participants had 2-fold higher PrEP uptake and adherence ($p<0.05$), with less frequent or severe IPV than control arm participants. The proposed study builds on these promising results and aims to evaluate the intervention in a fully powered cluster-randomized controlled trial across 22 administrative wards in Siaya County, Kenya, enrolling 72 AGYW per ward (total N=1,584). The primary objectives

(Aim 1) will be to test the effectiveness of the intervention on PrEP uptake and adherence immediately post-delivery (month 6 post- enrollment) and 6 months later (month 12). As secondary objectives we will test the intervention effect on IPV and relationship power (Aim 2). A rigorous process evaluation will explore mechanisms of change, contextual factors, and implementation considerations to inform future refinement and scale-up, using programmatic data, participant questionnaires, and qualitative interviews with participants and providers (Aim 3). The proposed study builds directly on our R34 intervention development work to develop the evidence base for this youth-designed, multi-level HIV prevention intervention. If effective, Tu'Washindi will be ideally positioned for sustainable integration into existing youth-focused programming to expand and support PrEP use in this priority population.

PUBLIC HEALTH RELEVANCE: The proposed study will test the effectiveness of our youth-designed, community-based intervention to address barriers to PrEP use faced by adolescent girls and young women in inequitable relationships and subject to intimate partner violence in Kenya. Combined with critical process data, these findings will fill a gap in the limited evidence base for interventions to expand PrEP uptake and adherence in this priority population and facilitate rapid translation from research to practice.

CRITIQUE 1

Significance: 2
Investigator(s): 1
Innovation: 3
Approach: 3
Environment: 1

Overall Impact: This is a revised R01, led by an Early Stage Investigator, to test the effectiveness of a youth-designed, multi-level, community-based intervention to address barriers to PrEP use faced by adolescent girls and young women in inequitable relationships and subject to intimate partner violence. The study will provide evidence regarding interventions to support uptake and sustained PrEP use amongst adolescent and young girls, as well as interventions to reduce intimate partner violence and improve experiences of relationship power. The study is informed by and aligned with a clearly articulated conceptual model, and responsive to findings from prior R34-supported work. It will be led by a highly qualified PI with the support of an exceptional team. The environments for research in Kenya as well as in the US are strong and conducive to the proposed work. The revised application is highly responsive to prior critiques. Overall, this is an excellent application. The main concern is the prior finding of low adherence to PrEP amongst intervention participants in the pilot study (though significantly higher than adherence in the control group). Regardless, if successful, there is potential for a high level of impact if this study is successfully implemented.

1. Significance:

Strengths

- Focuses on supporting sustained PrEP use amongst adolescents and young girls in a high HIV prevalence region of Kenya
- Addresses IPV and relationship power – this is particularly important in the study context where IPV and relationship power inequities are widespread
- Will provide evidence for a multi-level PrEP intervention involving AGYW, male partners of AGYW, and couples

Weaknesses

- None noted

2. Investigator(s):

Strengths

- The PI, Dr. Roberts, is an Epidemiologist and ESI with >15 years of research experience in the development and evaluation of HIV prevention strategies for women and girls in SSA. Dr. Roberts was the PI of the R34 in which the Tu'Washindi intervention was developed and tested.
- Dr. Agot is the site PI & has substantial experience and expertise in HIV prevention research and experience w/ research involving girls and women.
- Dr. Minnis is a well-qualified and highly experienced social epidemiologist and will focus on intervention delivery and quality assurance.
- Substantial expertise amongst investigators and technical advisors regarding HIV prevention, process evaluation, male engagement, clinical expertise, and evaluation of PrEP adherence biomarkers.

Weaknesses

- None noted.

3. Innovation:

Strengths

- If successful, would be one of the first to successfully address critical partner-related barriers to uptake and adherence and to directly involve male partners to build support for AGYW's PrEP use.

Weaknesses

- Limited methodologic innovation

4. Approach:

Strengths

- Builds on the PIs prior work which showed PrEP uptake and adherence 2x higher in the intervention arm compared to the control arm as well as less frequent and severe IPV
- Will assess PrEP uptake, adherence and persistence by measurement of TFVdp levels in dried blood spots at 6 and 12 months
- Includes a rigorous process evaluation to understand mechanisms of change and facilitate rapid translation of research to practice
- The intervention is youth-designed and tailored to meet the needs of AGYW
- The intervention is informed by and well-aligned with social cognitive theory in combination with socioecological framework for PrEP introduction.
- Incorporation of male engagement and couple's education were added to the design in response to formative research findings
- Pragmatic cRCT design is appropriate and a strength

- Will assess short- and long-term adherence with biomarker measurements of two active PrEP metabolites, TFVdp and emtricitabine triphosphate (FTCtp) to contribute a more thorough understanding of AGYW adherence behavior
- Pilot study – 97% retention over 6-month intervention duration; mean attendance in support sessions was 5.2 (out of 8 sessions); high level of participation in intervention components (>80%). High level of initiation (68% vs 32% in control arm) but low adherence (25% vs 13%)
- Plans to consult with community advisory boards is a strength
- Plans are described for monitoring intervention fidelity
- Includes a strong process evaluation, guided by the MRC guidelines for process evaluation of complex interventions

Weaknesses

- While adherence to PrEP (based on Wisepill openings) was low in the pilot study, adherence in the intervention arm was significantly higher than in the control arm (25% vs 13%). Though not optimal, the potential impact could have substantial public health impact. However, it seems that adherence could/should be clearly addressed in the planned support groups.
- How will pregnancy data be obtained?
- Self-reported STI data
- Partner data will not be collected
- Pre-piloting with 15-19 year

5. Environment:

Strengths

- Strong environment for the conduct of the proposed study

Weaknesses

- None noted

Study Timeline:

Strengths

- Timeline is comprehensive and reasonable – adequate time allocate for the activities including preparation, delivery, follow-up

Weaknesses

- None noted by reviewer

Protections for Human Subjects:

Acceptable Risks and/or Adequate Protections

Data and Safety Monitoring Plan (Applicable for Clinical Trials Only):

Acceptable

Inclusion Plans:

- Sex/Gender: Distribution justified scientifically
- Race/Ethnicity: Distribution justified scientifically
- For NIH-Defined Phase III trials, Plans for valid design and analysis: Not applicable
- Inclusion/Exclusion Based on Age: Distribution justified scientifically

Vertebrate Animals:
Not Applicable (No Vertebrate Animals)

Biohazards:
Not Applicable (No Biohazards)

Resubmission:
This resubmission is highly responsive to prior critiques.

Applications from Foreign Organizations:
Not Applicable (No Foreign Organizations)

Select Agents:
Not Applicable (No Select Agents)

Resource Sharing Plans:
Acceptable

Authentication of Key Biological and/or Chemical Resources:
Not Applicable (No Relevant Resources)

Budget and Period of Support:
Recommend as Requested

CRITIQUE 2

Significance: 2
Investigator(s): 4
Innovation: 3
Approach: 5
Environment: 3

Overall Impact: Revision of an R01 application from an early stage investigator in response to PA-20-144 and NOT-MH-20-035, that aims to test the effectiveness of a multilevel community-based intervention to increase uptake and adherence to oral PrEP among adolescent girls and young women

(AGYW) in Kenya. The PI has made substantial revisions to the proposal that are highly responsive to the prior critique, most notably perhaps, includes separating it entirely from the large DREAMS initiative upon which they were going to be basing the recruitment (and for which continued funding is in doubt) and positioning their recruiting to now better include adolescents. Approach is reasonable, though some minor weaknesses were identified that dampened enthusiasm, but it still has good potential to demonstrate the way forward for an important PREP- and IPV focused intervention for AGYW.

1. Significance:

Strengths

- Very significant need for data to guide PREP implementation for adolescent girls and young women (AGYM) in sub-Saharan Africa; the needs of AGYM in Kenya are reflective of the needs elsewhere in sub-Saharan Africa
- Do not currently have strong uptake of PREP in this setting, demonstrating the need for more effective interventions
- Intimate partner violence could also have significant impact on AGYM reproductive and sexual health decisions-making and risks.
- R34 of Tu-Washindi na PrEP (We're Winners with PREP) has significant pilot findings: PrEP uptake and adherence were two times higher in the intervention arm than in the control arm (each $p<0.05$), and intervention arm participants reported less frequent and severe IPV. (though mean age 22 years)

Weaknesses

- No weaknesses noted

2. Investigator(s):

Strengths

- Early stage PI appears well-prepared to do this work and has demonstrated success in this setting with pilot for this intervention and conduct of R34
- PI now spending slightly more time in Kenya (2, 2-week trips in the first year; 1 each year after that), which was suggested by prior reviewer and is appropriate
- Have now added a Kenyan pediatrician to the research team, which is helpful for both ensuring clinical expertise and for more rigorous involvement of local investigators.
- Robust epi and research team

Weaknesses

- Still have fairly minimal clinical expertise and Kenyan collaboration
- Would still like to see more PI time in-country given significant % effort and early stage investigator.
- Administration of a new study across 22 administrative wards and 1500+ participants to enroll warrants clear documentation of sufficient in-person effort for investigators.

3. Innovation:

Strengths

- Youth-centered design and adaptation to needs of adolescents and young women lacking in other intervention strategies for HIV prevention to-date
- Incorporating IPV/gender dynamics not often considered fully in combination with PREP uptake and adherence outcomes
- Innovative and rigorous methodology for adherence assessment in this setting with short- and long-term adherence measurements with biomarkers for two active PrEP metabolites

Weaknesses

- Specific components of intervention are not very novel, but important to have rigorous adaptation for this setting and population and for their onward scale-up

4. Approach:

Strengths

- Intervention and evaluation design focus on facilitating scalability, sustainability, and rapid adoption.
- Will test the effectiveness of youth-designed multilevel intervention to increase PrEP use and reduce IPV among Kenyan AGYW.
- PrEP being delivered through Ministry of Health clinical sites and will be accessible to all study participants. No changes to standard practice.
- Good case for moving away from use of Wisepill for adherence monitoring in this setting and for using alternate strategies for adherence measurement
- Still randomizing 22 administrative wards, N=1,584
- Thoroughly integrate YAB and CAB feedback now, as well as technical advisory committee
- Mixed methods process evaluation using implementation data, questionnaires, and qualitative IDIs, as well as project diary, is complex but rigorous. Appreciate measurement framework included.

Weaknesses

- Some questions about male partners participation or lack of participation remain. They do not include male partners, but there may still be some involvement or recognition of partners' participation. Have also provided pilot data suggesting male partner support
- Not clear the extent to which this study team will have capacity for such massive recruitment outside of DREAMS
- Explained why not testing for pregnancy within this study, but it is not clear whether data on pregnancy testing from MOH will be extracted in any form. This could have implications for IPV, adherence.
- Self-report for STI data not ideal
- Adherence measurement not ideal. Validation of measures for this population less clear.
- May be differences among potential study participants based on prior DREAMS exposure. Not clear that there will be exclusion if previously involved with DREAMS.

5. Environment:

Strengths
- Strong research infrastructure with RTI and partners in this setting in Kenya
- Appear to have ongoing critical support from county governments

Weaknesses
- Still have fairly minimal participation from Kenyan investigators

Study Timeline:
Strengths
- Realistic and well-considered

Weaknesses
- None noted

Protections for Human Subjects:
Acceptable Risks and/or Adequate Protections
- Well thought out plan for protecting vulnerable study participants

Data and Safety Monitoring Plan (Applicable for Clinical Trials Only):
Acceptable
- o Thorough and appropriate, includes DSMB

Inclusion Plans:
- Sex/Gender: Distribution justified scientifically
- Race/Ethnicity: Distribution justified scientifically
- For NIH-Defined Phase III trials, Plans for valid design and analysis: Not applicable
- Inclusion/Exclusion Based on Age: Distribution justified scientifically
- Recruitment targets appropriate to scientific aims.

Vertebrate Animals:
Not Applicable (No Vertebrate Animals)

Biohazards:
Not Applicable (No Biohazards)

Resubmission:
The PI has made substantial revisions to the proposal that are highly responsive to the prior critique, most notably perhaps, includes separating it entirely from the large DREAMS initiative upon which they were going to be basing the recruitment (and for which continued funding is in doubt) and positioning their recruiting to now better include adolescents.

Applications from Foreign Organizations:
Not Applicable (No Foreign Organizations)

Select Agents:
Not Applicable (No Select Agents)

Resource Sharing Plans:
Acceptable

- Detailed resource sharing plan

Authentication of Key Biological and/or Chemical Resources:
Acceptable

Budget and Period of Support:
Recommend as Requested

CRITIQUE 3

Significance: 2
Investigator(s): 1
Innovation: 1
Approach: 5
Environment: 1

Overall Impact: The proposed R01 proposal seeks to evaluate the efficacy of a multi-component intervention desired to increase PrEP uptake and adherence among adolescent girls and young women in Kenya. The scientific premise is solid with strong evidence highlighting the need to address HIV and IPV among this priority population. The PI is an early stage investigator who completed a promising R34 with strong pilot data that this R01 builds upon. The investigative team is exceptionally strong with a history of collaboration and requisite expertise to carry out the proposed activities. Strengths include the highly rigorous approach to the cluster RCT and process evaluation methods. This is a resubmission that was largely responsive to prior concerns. There remain two unaddressed weakness. First, the investigators make a compelling argument that male partners are the primary barrier to PrEP use and persistence (including a barrier to using the Wisepill device in the pilot). The investigators are not including the partners because of cost; however, they will receive an incentive as a “couple” for attending sessions. It would also seem viable to include them in the process evaluation at the very least in order to inform potential refinement. Second, Tu’ Washindi was piloted with 18-24 year olds. The investigators make a compelling argument for including 15-17 year olds but only briefly describe the pre-pilot testing and there no plans in place if the intervention is not deemed feasible or acceptable to the younger group. Additionally, greater details are needed on some of the measures, such as assessing pregnancy, partner HIV status, seroconversion across the study. These few weaknesses somewhat reduce enthusiasm for the proposal.

1. Significance:

Strengths

- HIV and IPV disproportionately impact adolescent girls and young women in Kenya.
- PrEP is an effective biomedical prevention strategy; however, uptake and persistence remain low among adolescent girls and young women in sub-Saharan Africa, which provides a strong scientific premise for interventions to address PrEP use among this priority population.
- IPV is highly prevalent and IPV and power inequities have been associated with HIV risk and linked to suboptimal PrEP adherence
- The proposed project builds on a promising pilot of Tu'Washindi that was designed in partnership with adolescent girls and young women in Kenya. The pilot was shown to be feasible, acceptable, and had promising trends on PrEP and IPV outcomes.

Weaknesses

- Partners are identified as a significant barrier to PrEP and the pilot data indicates the need for inclusion of partners. Thus, it is not clear why partners are not more integral to the intervention and why partner data is not collected.

2. Investigator(s):

Strengths

- PI Roberts is an exceptionally productive early stage investigator with over 15 years of experience in HIV prevention research with women and girls in sub-Saharan Africa, which has included obtaining extramural funding in the role PI and Co-I, and has a very strong publication track-record.
- The team also includes senior investigators, including epidemiologists, a biostatistician, as well as a very strong site PI who each have a wealth of experience focused on HIV prevention with women and girls, including studies focused on violence.
- The team is joined by a consultant (Dr. Digolo) who is a pediatrician and has over 15 years of experience working on studies focused HIV and gender-based violence among girls and young women in Kenya.
- The investigative team, including Consultant Digolo have a history of collaboration that informs the proposed project, including working together on the prior R34.

Weaknesses

- None noted.

3. Innovation:

Strengths

- This could be on the first interventions focused on PrEP and IPV among adolescent girls and young adults.
- The engagement of males and couples education based on formative research is important.
- The use of community based participatory research principles is notable strength.

Weaknesses

- None noted.

4. Approach:

Strengths

- The proposed large scale cluster RCT builds on a strong pilot data from an R34 grant that was developed in partnership with adolescent girls and young women and service providers in Kenya. The formative research led to a three-component intervention (empowerment-based group for adolescent girls and young adults, education events for couples, and community events about PrEP for the partners.
- The investigators propose a rigorous clustered RCT in which 22 administrative wards (outside of DREAMS) will be randomized to assess intervention effectiveness in on-going HIV prevention programs.
- A prospective process evaluation that triangulates different data sources will allow the investigators to understand intervention implementation and processes of change for potential future refinement and scalability.
- The inclusion of a range of stakeholders, including a youth advisory board and community advisory board throughout the project is notable strength.
- A strong justification is provided for the control clusters to be usual care services.
- Detailed intervention delivery and fidelity procedures and measures are provided.
- Good attention to study attrition is provided in proposed sample size and power calculation.
- The investigators describe potential alternative strategies in the context of COVID-19 and the risk of PrEP stock outs.

Weaknesses

- The preliminary studies report that participants may not have used the Wisepill device (only 25% of days in intervention condition) due to partner concerns (e.g., partner suspicion, violence). The proposed intervention is designed to address partner dynamics as “mechanisms of change.” This calls into question whether additional intervention activities/components with partners are needed to adequately address PrEP use among adolescent girls and young women in Kenya.
- The investigators propose to pre-test the activities with adolescents 15-17 because the majority of pilot participants were older in age. There are very few details provided on the pilot testing procedures and contingency plans if the Tu’ Washindi is not feasible or acceptable for this younger age group.
- While it may add some cost, it seems critical to collect some data from partners (even qualitative interviews to inform refinement) since the investigators describe how partners are the primary barrier to PrEP use.
- It seems that partners are included in some research activities (i.e., receiving an incentive to attend sessions)
- Pregnancy should be collected at each time point beyond relying on PrEP prescription of providers
- HIV seroconversion and partner HIV status should also be collected at each time point.

5. Environment:

Strengths

- The facilities at RTI, Impact Research Development Organization (IRDO), and the UNC CFAR provide strong environments for the proposed research.

Weaknesses

- None noted.

Study Timeline:

Strengths

- The timeline is detailed with adequate time for each of the planned activities

Weaknesses

- It is not clear from the timeline how long the pilot testing of the intervention with 15-17 years old will take and whether this will delay the timing of subsequent planned activities.

Protections for Human Subjects:

Acceptable Risks and/or Adequate Protections

Data and Safety Monitoring Plan (Applicable for Clinical Trials Only):

Acceptable

Inclusion Plans:

- Sex/Gender: Distribution justified scientifically
- Race/Ethnicity: Distribution justified scientifically
- For NIH-Defined Phase III trials, Plans for valid design and analysis: Not applicable
- Inclusion/Exclusion Based on Age: Distribution justified scientifically
- The study focuses on adolescent girls and young women which has strong scientific justification; however, the investigators make a strong argument for the need to include male partners given that they are the primary barrier to PrEP use among this priority population. It also seems that male partners may be involved in research activities since the couple will receive an incentive as part of attending a session.

Vertebrate Animals:

Not Applicable (No Vertebrate Animals)

Biohazards:

Not Applicable (No Biohazards)

Resubmission:

- The investigators were responsive to prior concerns. There remain a few concerns that did not provide strong justifications, specifically when male partners are involved (i.e., receiving

incentives for intervention activities) and limited details for how the intervention will be piloted with adolescent girls and contingency plans for refinements.

Applications from Foreign Organizations:
Not Applicable (No Foreign Organizations)

Select Agents:
Not Applicable (No Select Agents)

Resource Sharing Plans:
Acceptable

Authentication of Key Biological and/or Chemical Resources:
Not Applicable (No Relevant Resources)

Budget and Period of Support:
Recommend as Requested

THE FOLLOWING SECTIONS WERE PREPARED BY THE SCIENTIFIC REVIEW OFFICER TO SUMMARIZE THE OUTCOME OF DISCUSSIONS OF THE REVIEW COMMITTEE, OR REVIEWERS' WRITTEN CRITIQUES, ON THE FOLLOWING ISSUES:

PROTECTION OF HUMAN SUBJECTS: ACCEPTABLE

INCLUSION OF WOMEN PLAN: ACCEPTABLE

INCLUSION OF MINORITIES PLAN: ACCEPTABLE

INCLUSION ACROSS THE LIFESPAN: ACCEPTABLE

COMMITTEE BUDGET RECOMMENDATIONS: The budget was recommended as requested.

Footnotes for 1 R01 MH125671-01A1; PI Name: Roberts, Sarah Tyler

NIH has modified its policy regarding the receipt of resubmissions (amended applications).See Guide Notice NOT-OD-18-197 at https://grants.nih.gov/grants/guide/notice-files/NOT-OD-18-197.html. The impact/priority score is calculated after discussion of an application by averaging the overall scores (1-9) given by all voting reviewers on the committee and multiplying by 10. The criterion scores are submitted prior to the meeting by the individual reviewers assigned to an application, and are not discussed specifically at the review meeting or calculated into the overall impact score. Some applications also receive a percentile

ranking. For details on the review process, see http://grants.nih.gov/grants/peer_review_process.htm#scoring.
